# Extraction, Isolation, and Structural Characterization of Coconut Residue Polysaccharides: Immunomodulatory Activity, Antioxidant Capacity, and Gastrointestinal Digestive Behavior

**DOI:** 10.3390/foods15173105

**Published:** 2026-09-01

**Authors:** Phatthranit Klinmalai, SangGuan You, Nathdanai Harnkarnsujarit, Utoomporn Surayot

**Affiliations:** 1 Faculty of Agro-Industry, Chiang Mai University, Samut Sakhon 74000, Thailand; phatthranit.k@cmu.ac.th; 2 Cluster of Innovation for Sustainable Seafood Industry and Value Chain Management, Chiang Mai University, Samut Sakhon 74000, Thailand; 3 Department of Marine Convergence Science, Kangwon National University, Gangneung, Gangwon 25457, Republic of Korea; umyousg@kangwon.ac.kr; 4 East Coast Life Sciences Institute, Kangwon National University, Gangneung, Gangwon 25457, Republic of Korea; 5 Department of Packaging and Materials Technology, Faculty of Agro-Industry, Kasetsart University, 50 NgamWong Wan Rd., Latyao, Chatuchak, Bangkok 10900, Thailand; nathdanai.h@ku.ac.th; 6 Center for Advanced Studies for Agriculture and Food (CASAF), Kasetsart University Institute for Advanced Studies (KUIAS), Kasetsart University, 50 Ngam Wong Wan Rd., Latyao, Chatuchak, Bangkok 10900, Thailand

**Keywords:** coconut residue, polysaccharides, immunomodulatory, gastrointestinal digestion, macrophage activation, radical-scavenging activity, valorization

## Abstract

Coconut residue, an abundant agro-industrial by-product, represents a potential source of bioactive polysaccharides. In this study, crude polysaccharides (CP0) were extracted from coconut residue and sequentially purified by DEAE–Sepharose Fast Flow anion-exchange chromatography and Sepharose CL-6B gel-filtration chromatography, yielding two major fractions, CP1 and CP2. Structural characterization revealed galactose- and glucose-rich heteropolysaccharides with distinct molecular characteristics. CP0 exhibited a molecular weight of 369.2 kDa, while the purified fractions CP1 and CP2 had molecular weights of 455.8 and 72.6 kDa, respectively. All fractions contained high carbohydrate contents (96.7–97.7%) and low protein contents (2.30–2.90%). Among the fractions, CP2 exhibited the greatest in vitro radical-scavenging capacity in DPPH and ABTS assays and stimulated the highest NO production in RAW264.7 macrophages without reducing cell viability. Simulated gastrointestinal digestion of CP2 was associated with a progressive reduction in apparent molecular weight and slight changes in relative monosaccharide composition. Gastric- and intestinal-digested CP2 preparations generally exhibited greater in vitro radical-scavenging capacity than native CP2. Intestinal-digested CP2 also produced the highest NO response among the recovered preparations and was associated with increased expression of iNOS, TNF-α, IL-1β, and IL-6 and reduced IL-10 expression, indicating a predominantly pro-inflammatory macrophage response. These biological responses occurred concurrently with digestion-associated molecular changes; however, the present experimental design does not establish a direct causal structure–activity relationship. Overall, the findings provide new information on the physicochemical characteristics, gastrointestinal behavior, radical-scavenging capacity, and macrophage responses of purified coconut residue polysaccharides and support further investigation of CP2 for potential food-related applications and value-added utilization of coconut residue.

## 1. Introduction

Coconut (*Cocos nucifera* L.), a perennial plant belonging to the family Arecaceae, is widely cultivated throughout tropical and subtropical regions, including Central and South America, Africa, South and Southeast Asia, and the Pacific Islands [1]. In Thailand, coconut is considered one of the country’s most economically important perennial crops and plays a significant role in local livelihoods, traditional cuisine, cosmetics, medicine, and agro-industrial production. Coconut fruits are extensively processed into a variety of commercial products, including fresh coconut, coconut milk, coconut cream, and virgin coconut oil [2]. However, the large-scale processing of these products generates substantial quantities of coconut residue as a by-product. The increasing generation of this residue highlights the need for sustainable valorization strategies that can transform an underutilized biomass into high-value functional and bioactive ingredients rather than treating it solely as an agro-industrial waste stream.

Coconut residue, also referred to as coconut by-product or coconut waste, is the solid material remaining after extraction processes. Although often underutilized, it is a valuable source of nutrients and bioactive compounds, including dietary fiber, proteins, vitamins, residual lipids, and polysaccharides [3,4]. Consequently, the valorization of coconut residue has attracted increasing interest as a sustainable approach to reduce agro-industrial waste while generating value-added functional ingredients. In recent years, polysaccharides derived from coconut kernel, coconut water, coconut husk, and coconut residue have received considerable attention due to their diverse biological activities and potential health benefits [5,6,7,8]. These polysaccharides have been reported to possess antioxidant, antimicrobial, anti-inflammatory, anticancer, anti-angiogenic, antiproliferative, and immunomodulatory properties, highlighting their potential applications in nutraceuticals and functional foods. Importantly, the biological activities of plant-derived polysaccharides are strongly associated with their molecular characteristics, including molecular weight, monosaccharide composition, branching pattern, degree of polymerization, and other structural features. Therefore, the biological potential of coconut residue polysaccharides may depend not only on their intrinsic composition but also on the extraction, isolation, and purification processes used to obtain the polysaccharide fractions. These processes can selectively recover different polysaccharide populations and consequently influence their structural characteristics and functional properties.

In the food industry, plant-derived polysaccharides are widely utilized as functional ingredients because of their technological and physiological properties [9]. Coconut residue polysaccharides have attracted interest as potential sources of dietary fiber and bioactive ingredients for food-related applications [9,10,11]. Previous studies have reported that crude polysaccharides extracted from defatted coconut residue can stimulate probiotic proliferation and acidification in vitro [9], suggesting potential prebiotic properties that warrant further investigation. However, such effects may depend on polysaccharide composition, molecular characteristics, and gastrointestinal behavior. Moreover, crude extracts contain heterogeneous populations of polysaccharides and other co-extracted components, which may influence the properties attributed to individual polysaccharide fractions. Therefore, isolation and purification are important for characterizing distinct polysaccharide fractions and examining associations between their physicochemical characteristics and biological responses. Although prebiotic activity and colonic fermentation were not evaluated in the present study, these aspects represent important directions for future investigation of purified coconut residue polysaccharides.

Despite the growing interest in coconut-derived polysaccharides, their immunomodulatory potential remains largely unexplored. Most previous studies have focused on coconut kernel oil using animal models [12,13] or coconut shell proteins in macrophages [14], whereas limited information is available regarding the effects of coconut residue-derived polysaccharides on immune responses in macrophage cells. Furthermore, similar to many plant-derived polysaccharides, their high molecular weight and structural complexity may limit biological activity by reducing solubility, bioavailability, and cellular uptake. Consequently, structural modification of isolated polysaccharides is of particular interest because alterations in molecular size and structural organization may modify their solubility, accessibility, cellular interaction, and ultimately biological activity. Among the available modification approaches, gastrointestinal digestion is especially relevant to food applications because polysaccharides intended for functional foods and nutraceuticals are inevitably exposed to the gastrointestinal environment. Therefore, understanding how the molecular characteristics and biological properties of isolated coconut residue polysaccharides change under simulated gastrointestinal conditions is important for evaluating their digestive behavior and associated biological responses.

In vitro gastrointestinal digestion is widely used to evaluate the stability, bioactivity, and bioavailability of polysaccharides because of its simplicity, rapidity, and reproducibility [15,16]. This process involves enzymatic and physicochemical reactions that degrade macromolecules, thereby enhancing nutrient accessibility and potentially improving biological activity. Previous studies have shown that gastrointestinal digestion can reduce the molecular weight of plant-derived polysaccharides [17,18], which may contribute to enhanced immunoregulatory effects. Accordingly, gastrointestinal digestion can be considered not only an evaluation of digestive behavior but also a physiologically relevant structural modification process. The resulting changes in molecular weight, structural characteristics, and physicochemical properties may provide information on associations between molecular characteristics and biological responses of polysaccharides and help explain changes in their antioxidant and immunomodulatory activities. However, the extent to which isolation and subsequent digestion-induced structural modification influence the biological properties of coconut residue polysaccharides remains insufficiently understood.

Although previous studies have investigated the extraction, composition, antioxidant activity, and prebiotic potential of coconut-derived polysaccharides, limited information is available on how purified coconut residue polysaccharide fractions respond to gastrointestinal digestion and whether digestion-associated molecular changes are accompanied by changes in their biological activities. Moreover, previous studies of digestion-associated molecular changes in polysaccharide bioactivity have predominantly focused on polysaccharides from other plant or fungal sources, leaving purified coconut residue polysaccharides comparatively underexplored. Therefore, the present study aimed to isolate and purify polysaccharides from coconut residue, compare the physicochemical characteristics and biological activities of the resulting fractions, and further investigate the gastrointestinal behavior of the selected bioactive fraction. Changes in molecular weight and monosaccharide composition during simulated gastrointestinal digestion were evaluated together with antioxidant activity and macrophage responses before and after digestion. By integrating fraction-level characterization with post-digestion biological evaluation, this study provides new information on the associations among the molecular characteristics, gastrointestinal behavior, and functional properties of coconut residue polysaccharides.

## 2. Materials and Methods

### 2.1. Sample Preparation

Coconut residue was obtained from a local coconut-processing facility in Samut Sakhon, Thailand, where it was generated as a by-product of coconut water processing. The residue was collected in November 2024 from a single production batch. Information regarding the specific coconut cultivar and fruit maturity stage was not available from the processing facility. The sample was thoroughly rinsed with distilled water, dried at 40 °C for 24 h, and milled to pass through an 80-mesh sieve. The dried residue had an initial moisture content of approximately 7%. The proximate composition of the dried coconut residue, expressed on a dry-weight basis, was 5.91% crude protein, 16.13% crude fat, and 2.47% ash. The remaining 75.48% was calculated by difference and represented the combined carbohydrate and fiber fraction. The prepared powder was packed in sealed polyethylene bags and stored at −20 °C until further analysis.

### 2.2. Polysaccharide Extraction

Coconut residue powder (200 g) was first defatted, and low-molecular-weight compounds were removed by soaking in 80% (*v*/*v*) ethanol for 24 h. The defatted coconut residue powder was extracted with 1000 mL of distilled water at a solid-to-liquid ratio of 1:5 (*w*/*v*) using an autoclave at 121 °C for 30 min. Following extraction, insoluble materials were removed by centrifugation, and the resulting supernatant was concentrated to approximately 200 mL using a rotary evaporator.

Polysaccharides were precipitated by adding ethanol to a final concentration of 85% (*v*/*v*) and incubating at 4 °C overnight. The precipitate was collected, washed with acetone, and deproteinized using 3% (*w*/*v*) trichloroacetic acid (TCA), followed by the Sevag method. The resulting crude polysaccharide (CP0) was freeze-dried and stored in a sealed container until further purification and analysis.

### 2.3. Isolation and Purification of Polysaccharides

CP0 was first fractionated by anion-exchange chromatography using a DEAE–Sepharose Fast Flow column (GE Healthcare Bio-Science AB, Uppsala, Sweden). CP0 was applied to the column at a concentration of 10 mg/mL and sequentially eluted with distilled water followed by NaCl solutions (0.5–2.0 M). Carbohydrate-containing fractions were monitored using the phenol–sulfuric acid method at 490 nm. The major neutral polysaccharide fraction eluted with distilled water was collected for further purification.

The neutral fraction was subsequently purified by gel-filtration chromatography using a Sepharose CL-6B column (1.5 × 50 cm; bed volume approximately 88 mL). The sample was loaded at 20 mg/mL with a loading volume of 5 mL (100 mg in total) and eluted with 10 mM phosphate buffer (pH 7.0) at a flow rate of 0.5 mL/min. Fractions were collected at 5 mL per tube, and carbohydrate-containing fractions were monitored using the phenol–sulfuric acid method at 490 nm. Fractions corresponding to the two major chromatographic peaks were pooled separately according to the peak boundaries, with tubes 50–100 designated as CP1 and tubes 150–200 designated as CP2. The pooled fractions were dialyzed against distilled water using dialysis membranes with a molecular-weight cut-off (MWCO) of 3.5 kDa for 72 h, with periodic replacement of the dialysis water, and subsequently freeze-dried. Chromatographic purification was performed in three independent runs under identical conditions, and the corresponding fractions from each run were processed separately.

### 2.4. Chemical Composition Analysis

The chemical composition of the sample, including carbohydrates, proteins, and sulfates, was assessed using standardized references: D-glucose for carbohydrates, BSA for proteins, and K_2_SO_4_ for sulfates. To perform the analysis, various methods were applied: the phenol-sulfuric acid technique [19] for carbohydrates, the Folin–Ciocalteu method [20] for proteins, the BaCl_2_ gelatin method [21] for sulfates, and a sulfamate/m-hydroxydiphenyl assay for uronic acid content using glucuronic acid as the standard [22].

For monosaccharide composition analysis, polysaccharide samples (2 mg) were hydrolyzed with 4 M trifluoroacetic acid (TFA) at 100 °C for 6 h. The hydrolysate was dried under a nitrogen stream, reduced with sodium borodeuteride (NaBD_4_), and neutralized with glacial acetic acid. The sample was subsequently washed with methanol and acetylated using acetic anhydride at 100 °C. The resulting derivatives were extracted with methylene chloride, dried over anhydrous sodium sulfate, and analyzed by gas chromatography–mass spectrometry (GC–MS) (6890N/MSD5973, Agilent Technologies, Santa Clara, CA, USA) equipped with an HP-5MS capillary column.

### 2.5. Structure Analysis

#### 2.5.1. Molecular Weight (Mw) Determination

The molecular characteristics of the polysaccharides were determined by high-performance size-exclusion chromatography coupled with ultraviolet, multi-angle laser light scattering, and refractive-index detectors (HPSEC–UV–MALLS–RI). Briefly, polysaccharide samples (5–10 mg) were dissolved in 2 mL of distilled water, heated at 60 °C for 10 min with intermittent vortex mixing to ensure complete dissolution, and filtered through a 3 µm filter prior to analysis. Separation was performed using a TSK-G5000PW column (7.5 × 600 mm; Toso Biosep, Montgomeryville, PA, USA) with 0.15 M NaNO_3_ containing 0.02% NaN_3_ as the mobile phase at a flow rate of 0.4 mL/min. The column was coupled to UV, MALLS, and RI detectors, with the RI detector maintained at 40 °C.

Molecular parameters were calculated using ASTRA 6.1 software (Wyatt Technology Corp., Santa Barbara, CA, USA) based on the light-scattering and refractive-index signals. A refractive-index increment (dn/dc) of 0.146 mL/g was used for the polysaccharide samples. Bovine serum albumin (BSA; dn/dc = 0.185 mL/g) was analyzed as a system-performance reference prior to sample analysis. Light-scattering data were processed using the Debye model. The weight-average molecular weight (Mw), number-average molecular weight (Mn), radius of gyration (Rg), and polydispersity index (PDI = Mw/Mn) were obtained from the resulting molecular-weight distributions. The reported molecular parameters were derived from repeated injections of the prepared samples.

#### 2.5.2. Fourier Transform Infrared Spectroscopy (FT-IR) Analysis

The FT-IR spectra of the polysaccharide fractions were recorded using a Tensor 27 spectrophotometer (Bruker, Bremen, Germany). Each sample was mixed with potassium bromide (KBr), pressed into a pellet, and analyzed within the spectral range of 4000–400 cm^−1^ at a resolution of 4 cm^−1^ with 32 scans per spectrum.

#### 2.5.3. Methylation Analysis

Glycosidic linkage patterns were characterized by methylation analysis following a modified method. Prior to methylation, uronic acid residues were reduced to their corresponding neutral forms. The polysaccharide sample (2–3 mg) was dissolved in dimethyl sulfoxide (DMSO) and methylated using methyl iodide. The fully methylated product was subsequently hydrolyzed with 4 M trifluoroacetic acid (TFA) at 100 °C for 6 h [23]. The resulting hydrolysate was reduced with sodium borodeuteride (NaBD_4_) and acetylated using acetic anhydride. The derivatized products were extracted with methylene chloride, and the partially methylated alditol acetates (PMAAs) were analyzed by GC–MS.

### 2.6. Digestion In Vitro

#### 2.6.1. Saliva Digestion

In vitro saliva digestion was conducted following the methodology outlined by Wang et al. (2018) [16] with minor modifications. Briefly, CP2 solution (20 mL, 3 mg/mL) was mixed with 6 mL of artificial saliva fluid containing KCl, KSCN, NaH_2_PO_4_, Na_2_SO_4_, NaCl, NaHCO_3_, urea, and α-amylase. The pH of the mixture was adjusted to 6.8 using 0.1 M HCl and diluted with 40 mL of distilled water. The digestion mixture was incubated in a thermostatic shaker at 37 °C for 5, 15 and 30 min. Following digestion, an aliquot (10 mL) was transferred to a boiling water bath for 5 min to terminate enzyme activity. After cooling, the solution was neutralized, centrifuged at 8000× *g* and 4 °C for 10 min, and the supernatant was collected. The obtained supernatant was dialyzed against distilled water and subsequently freeze-dried for further analysis.

#### 2.6.2. Simulated Gastric Digestion

Simulated gastric digestion was carried out according to the method of Tedeschi et al. (2009) [24] with slight modifications. The gastric electrolyte solution was prepared by dissolving NaCl (620 mg), KCl (220 mg), CaCl_2_·2H_2_O (300 mg), and NaHCO_3_ (120 mg) in 200 mL of distilled water. The pH was adjusted to 3.0 using 1.0 M HCl. Lipase from *Rhizopus oryzae* (50 mg; Cat. No. 80612; specific activity ≥ 30 U/mg; Sigma-Aldrich, St. Louis, MO, USA) and pepsin from porcine gastric mucosa (47.2 mg; Cat. No. P7012; specific activity ≥ 2500 U/mg protein; Sigma-Aldrich, St. Louis, MO, USA) were added, followed by 2 mL of 1.0 M acetic acid (pH 5.0), to prepare the simulated gastric juice.

The salivary-digested CP2 was mixed with simulated gastric juice to obtain a final concentration of 4.0 mg/mL. The control group consisted of simulated gastric juice without sample addition. The mixtures were incubated in a shaking water bath at 37 °C and 100 rpm. Aliquots (5 mL) were collected after 5, 15, 30, 60, and 120 min, heated in boiling water for 10 min to terminate digestion, and neutralized with 1.0 M NaHCO_3_.

#### 2.6.3. Simulated Small Intestinal Digestion

Simulated small intestinal digestion was performed based on the method of Tedeschi et al. (2009) [24] with minor modifications. The intestinal electrolyte solution was prepared by dissolving NaCl (270 mg), KCl (32.5 mg), and CaCl_2_·2H_2_O (16.5 mg) in 50 mL of distilled water, followed by adjustment of pH to 7.0 using 1.0 M NaHCO_3_. Subsequently, 50 mL of 7% (*w*/*v*) pancreatin solution prepared from pancreatin from porcine pancreas (Cat. No. P7545, 8 × USP specifications; Sigma-Aldrich, St. Louis, MO, USA), trypsin from porcine pancreas (6.5 mg; Cat. No. T4799, ≥10,000 BAEE units/mg protein; Sigma-Aldrich, St. Louis, MO, USA), and 100 mL of 4% (*w*/*v*) bile salt solution were added to prepare the simulated intestinal juice.

After 2 h of simulated gastric digestion, the gastric digest was neutralized with 1.0 M NaHCO_3_ and mixed with the intestinal juice at a ratio of 10:3 (*v*/*v*). The resulting mixture was incubated at 37 °C for simulated intestinal digestion. Samples were collected at 0, 15, 30, 60, and 120 min and immediately heated in boiling water for 10 min to terminate enzymatic activity.

### 2.7. Assessment of Macrophage-Stimulating Activity

#### 2.7.1. Cell Line and Cell Culture

The RAW264.7 cell line was obtained from the Korean Cell Line Bank (Seoul, Republic of Korea). The cells were cultured in Roswell Park Memorial Institute medium (RPMI, Gibco, Grand Island, NY, USA) supplemented with 10% (*v*/*v*) fetal bovine serum (FBS) and 1% antibiotics (streptomycin and penicillin) at 37 °C in a humidified incubator containing 5% CO_2_.

#### 2.7.2. Assay of Cell Viability

Cytotoxicity was assessed using a WST assay with the EZ-Cytox assay kit (Daeil Lab Service, Seoul, Republic of Korea). The culture medium served as a control, while lipopolysaccharide (LPS) was used as a positive control due to its potent immunostimulatory properties. Cells were seeded in 96-well plates at a density of 1 × 10^6^ cells/mL and treated with polysaccharides at varying concentrations (100, 200, 400 μg/mL), LPS (1 μg/mL), or culture medium alone for 24 h. Following incubation, the medium was removed, and 110 μL of WST solution was added to each well, followed by further incubation for 1 h. Absorbance was recorded at 450 nm using a microplate reader (BioTek, Shoreline, WA, USA). Cell viability (%) was calculated using the following formula:

Cell viability (%) = [Absorbance of sample/Absorbance of control] × 100

#### 2.7.3. Measurement of Nitric Oxide (NO) Production

Cells (1 × 10^6^ cells/mL) were cultured in 96-well plates overnight, and then cells were treated with varying concentrations of polysaccharides (100, 200, 400 μg/mL) or LPS (1 μg/mL) for 24 h. After incubation, the culture medium (100 μL) was transferred to a new 96-well plate, followed by the addition of an equal volume of Griess reagent (Promega, USA) to each well. The plates were incubated at room temperature for 10 min before being recorded with a microplate reader at 540 nm. The concentration of NO was calculated using a standard curve established with various amounts of sodium nitrite (NaNO_2_).

#### 2.7.4. Endotoxin Test

Endotoxin contamination in the polysaccharide samples was evaluated using the Limulus amebocyte lysate (LAL) assay with a commercial endotoxin detection kit (ETOXATE™, Sigma-Aldrich, St. Louis, MO, USA) according to the manufacturer’s instructions. Sterile endotoxin-free water was used for the preparation and dilution of the polysaccharide samples and endotoxin standard. Briefly, each sample or endotoxin standard solution was placed in a sterile endotoxin-free glass tube, followed by the addition of 100 µL of LAL working reagent. The mixtures were incubated at 37 °C for 1 h and subsequently examined for gel formation. Formation of a firm gel was interpreted as a positive result for detectable endotoxin, whereas the absence of firm gel formation was interpreted as a negative result. All measurements were performed in triplicate.

#### 2.7.5. Quantitative Analysis of mRNA Expression

Cells (1 × 10^6^ cells/mL) were cultured in 24-well plates and exposed to several concentrations of polysaccharides (100–400 μg/mL) for 24 h. Total RNA was extracted from the treated cells using TRI Reagent^®^ (Molecular Research Center, Cincinnati, OH, USA) and subsequently reverse-transcribed into complementary DNA (cDNA) using a high-capacity cDNA reverse transcription kit (Thermo Fisher Scientific, Waltham, MA, USA). Real-time PCR was performed using TB Green^®^ Premix Ex Taq™ II (Takara Bio Inc., Kusatsu, Japan) on the QuantStudio™ 3 Flex system (Applied Biosystems, Waltham, MA, USA). β-Actin was used as the reference gene for normalization of the expression levels of IL-1β, IL-6, IL-10, TNF-α, and iNOS. The primer sequences, accession numbers, amplicon lengths, and annealing temperatures for all target genes and the reference gene (β-actin) are provided in Supplementary Table S1. Relative gene expression was calculated using the 2^−ΔΔCt^ method [25].

### 2.8. Antioxidant Activity Assay

#### 2.8.1. Assay of DPPH Radical Scavenging Ability

DPPH radical scavenging activity was determined according to Delattre et al. [26]. Sample solutions (0–1.6 mg/mL) were mixed with an equal volume of 0.1 mM DPPH in ethanol. The mixtures were incubated in the dark at 25 °C for 30 min, and absorbance was measured at 517 nm using a microplate reader. Vitamin C (Vc) was used as a positive control. The scavenging activity (%) was calculated as:

Scavenging activity (%) = [(A control − A sample)/A control] × 100

where A sample represents the absorbance at 517 nm of the mixture: 1 mL of polysaccharide solution + 1 mL of DPPH (0.1 mM in ethanol); and A control represents the absorbance at 517 nm of the mixture: 1 mL of ultrapure water + 1 mL of DPPH (0.1 mM in ethanol).

#### 2.8.2. Assay of ABTS Radical Scavenging Ability

ABTS radical scavenging activity was evaluated as previously described [27]. The ABTS radical was generated by mixing ABTS (7 mM) with potassium persulfate (2.45 mM) and incubating in the dark for 16–18 h. The solution was diluted with 70% ethanol to an absorbance of 0.70 ± 0.05 at 734 nm. CP solution (0.2 mL) was mixed with 2 mL of ABTS solution, incubated for 20 min, and absorbance was measured at 734 nm. Vitamin C (Vc) was used as a positive control.

#### 2.8.3. Superoxide Radical Scavenging Activity

Superoxide radical scavenging activity was determined according to Shang et al. [28] with slight modifications. Sample solutions were mixed with Tris–HCl buffer (50 mM, pH 8.2) and incubated at 25 °C for 20 min. Pyrogallol (25 mM) was then added, and the reaction was allowed to proceed for 4 min before being terminated with 8 mM HCl. Absorbance was measured at 325 nm using a microplate reader. Vitamin C (Vc) was used as a positive control. The scavenging activity (%) was calculated using the same equation as above.

### 2.9. Statistical Analysis

Data are presented as mean ± standard deviation (SD) from triplicate measurements (*n* = 3). Normality and homogeneity of variance were assessed before statistical analysis. Data involving treatment and concentration were analyzed using two-way ANOVA followed by Tukey’s multiple-comparison test, while gene-expression data were analyzed using one-way ANOVA followed by Tukey’s test. A *p*-value < 0.05 was considered statistically significant.

## 3. Results

### 3.1. Isolation and Purification and Chemical Composition of Polysaccharide

**Figure 1:**
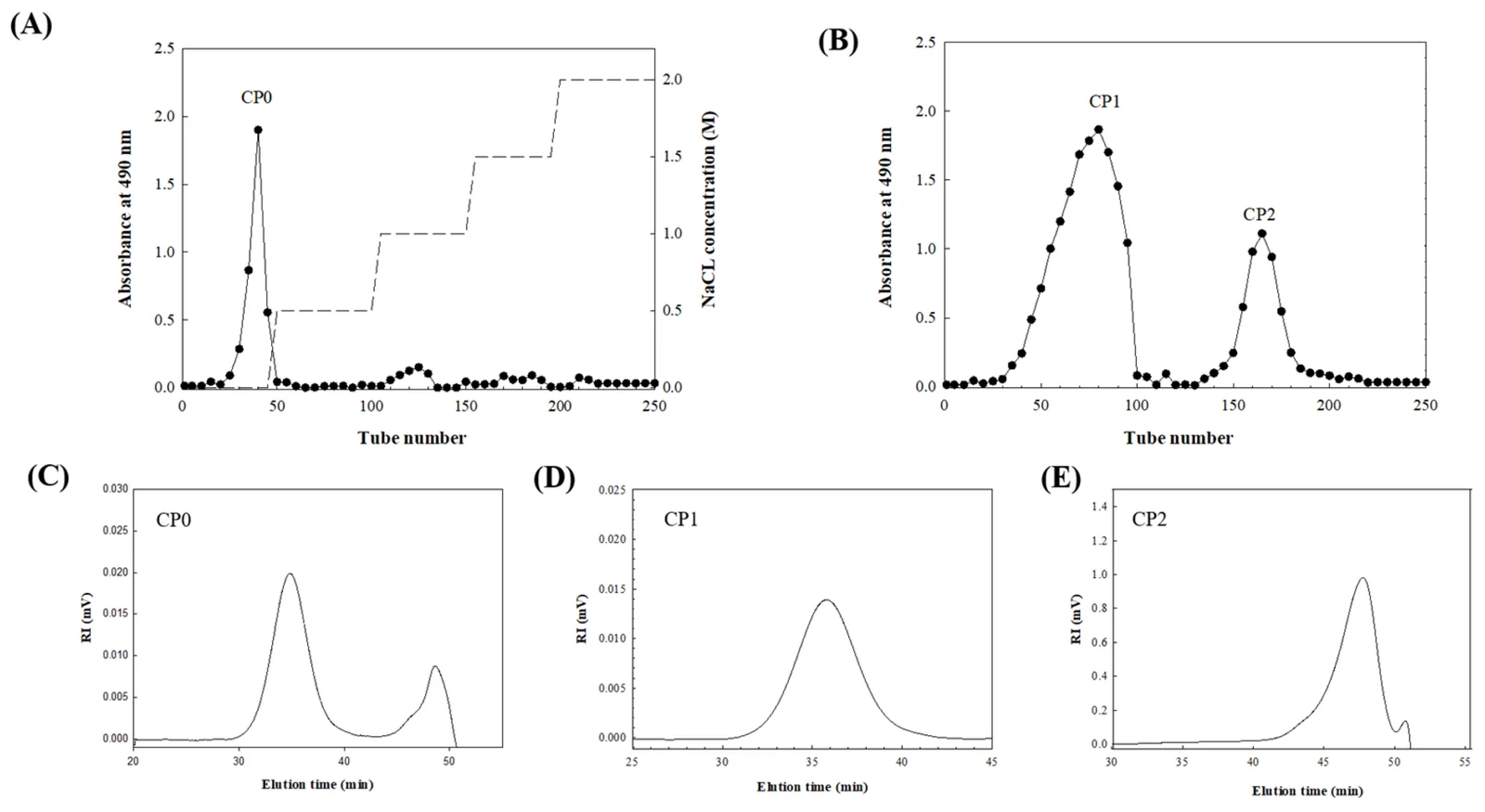
Purification of coconut residue polysaccharides by anion-exchange and gel filtration chromatography. (**A**) Elution profile of crude polysaccharide (CP0) on a DEAE–Sepharose Fast Flow column using distilled water and stepwise NaCl elution (0.5–2.0 M). (**B**) Elution profile of CP0 on a Sepharose CL-6B column showing the separation of two major fractions (CP1 and CP2). (**C**) HPSEC chromatogram of crude polysaccharide (CP0). (**D**) HPSEC chromatogram of purified fraction CP1. (**E**) HPSEC chromatogram of purified fraction CP2.

**Scheme 1:**
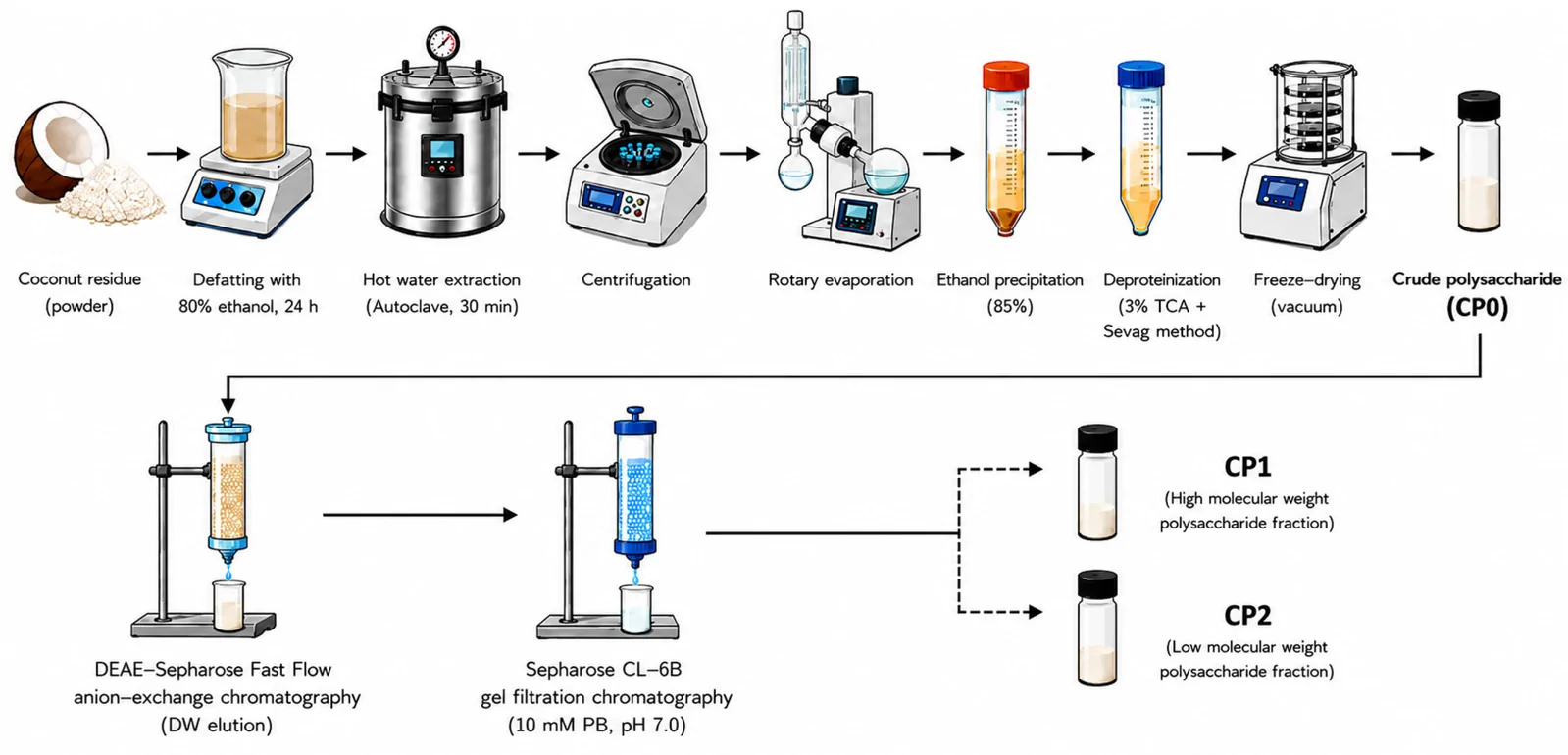
The extraction and purification procedure for coconut residue polysaccharides.

Crude polysaccharides (CP0) were successfully extracted from coconut residue with a recovery yield of 5.72%. The extraction and purification workflow is illustrated in Scheme 1, while the chromatographic profiles are presented in Figure 1A,B. Fractionation of CP0 by DEAE–Sepharose Fast Flow anion-exchange chromatography yielded a major neutral polysaccharide fraction eluted with distilled water, accounting for approximately 95% of the recovered polysaccharide material (Figure 1A). Subsequent purification of the neutral fraction by Sepharose CL-6B gel-filtration chromatography resolved two major polysaccharide fractions, designated CP1 and CP2 (Figure 1B), which represented approximately 67% and 33%, respectively, of the total polysaccharide material recovered from the two pooled fractions. The CP0 extraction yield obtained in this study was higher than those previously reported for coconut-derived polysaccharides [9,29,30].

The chemical compositions of CP0, CP1, and CP2 are summarized in Table 1. CP0 contained 96.7% carbohydrate and 2.90% protein, indicating a high polysaccharide content. Following purification, CP1 and CP2 exhibited slightly higher carbohydrate contents of 97.1% and 97.7%, respectively, while their protein contents remained low at 2.90% and 2.30%, respectively. These results indicate that the chromatographic purification process effectively yielded polysaccharide-rich fractions with relatively low residual protein contents. Monosaccharide analysis (Figure S1) showed that CP0, CP1, and CP2 were predominantly composed of galactose and glucose, although their relative proportions differed after purification. Notably, CP2 exhibited a higher galactose-to-glucose ratio than CP1, indicating that chromatographic purification effectively separated polysaccharides with distinct monosaccharide compositions. These compositional differences occurred concurrently with differences in molecular weight and glycosidic linkage profiles; however, their individual contributions to the observed biological activities cannot be established from the present data. Similar galactose- and glucose-rich polysaccharides have been reported from coconut kernel [9], whereas polysaccharides isolated from coconut pulp residue contain higher proportions of fructose and xylose [31]. These findings suggest that the structural diversity of coconut-derived polysaccharides may be influenced by the botanical source and extraction and purification procedures, resulting in differences in monosaccharide composition and physicochemical characteristics [32].

### 3.2. Molecular Weight Characteristics and Homogeneity of Purified Polysaccharide Fractions (CP1 and CP2)

The HPSEC chromatograms of CP0, CP1, and CP2 are presented in Figure 1C–E, and their molecular weight parameters are summarized in Table 2. The chromatogram of CP0 exhibited two distinct peaks, indicating the presence of polysaccharide populations with different molecular sizes. Following chromatographic fractionation, CP1 and CP2 each exhibited a single dominant peak, confirming the separation of two fractions with distinct molecular weight distributions.

CP0 exhibited an average molecular weight (Mw) of 369.2 kDa, a radius of gyration (Rg) of 58.8 nm, and a polydispersity index (PDI) of 1.52. CP1 showed the highest Mw (455.8 kDa) and the broadest molecular weight distribution (PDI = 2.12), indicating substantial molecular-weight heterogeneity despite the presence of a single dominant chromatographic peak. In contrast, CP2 exhibited a substantially lower Mw (72.6 kDa) and a lower PDI (1.35), indicating a narrower molecular weight distribution than CP0 and CP1.

Interestingly, CP2 exhibited a larger Rg (64.5 nm) than CP1 (54.7 nm) despite its considerably lower Mw. Because Rg reflects the spatial distribution of polymer mass and can be influenced by molecular architecture, branching, aggregation, and solution behavior, this observation alone is insufficient to establish a specific molecular conformation [33]. Therefore, the present results demonstrate that chromatographic fractionation separated polysaccharide fractions with distinct Mw, Rg, and molecular weight distributions, while further conformation-sensitive analyses would be required to clarify their detailed molecular organization.

### 3.3. FT-IR Spectra

**Figure 2:**
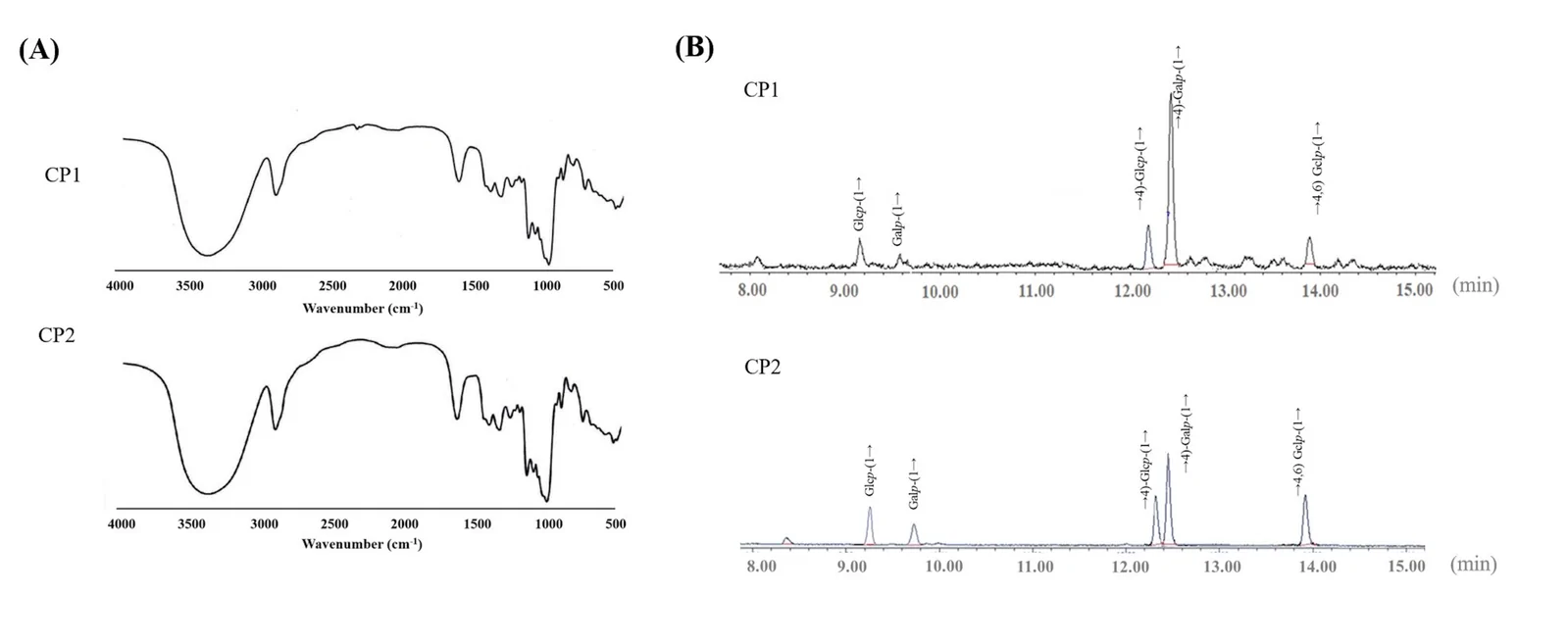
Structural characterization of coconut residue polysaccharides. (**A**) FT-IR spectra of purified fractions CP1 and CP2. (**B**) Representative GC–MS chromatograms of partially methylated alditol acetates (PMAAs) derived from CP1 and CP2 for glycosidic linkage analysis.

The FT-IR spectra of CP1 and CP2 are presented in Figure 2A. Both fractions exhibited similar spectral profiles, indicating comparable major functional-group characteristics. A broad absorption band at approximately 3400 cm^−1^ was attributed to the stretching vibration of hydroxyl (O–H) groups, while the absorption band near 2930 cm^−1^ corresponded to C–H stretching vibrations of aliphatic groups. A weak band around 1640 cm^−1^ was assigned to the bending vibration of absorbed water molecules. The fingerprint region (1200–900 cm^−1^) displayed characteristic absorption bands associated with carbohydrate structures. In particular, the bands observed at approximately 1100–1030 cm^−1^ were assigned to C–O–C and C–O stretching vibrations associated with glycosidic linkages and pyranose ring structures, supporting the polysaccharide nature of both fractions [34]. Overall, these spectral features indicate similarities in the major functional groups of CP1 and CP2. However, FT-IR analysis alone is insufficient to establish their detailed primary structures, branching architectures, or molecular conformations, which would require complementary structural analyses such as NMR spectroscopy.

### 3.4. Glycosidic Linkages Analysis

To elucidate the glycosidic linkage patterns of CP1 and CP2, methylation analysis was performed, followed by hydrolysis, reduction, acetylation, and GC–MS analysis of partially methylated alditol acetates (PMAAs). The resulting chromatograms and linkage compositions are presented in Figure 2B, S2 and S3, and Table 3. Five major glycosidic linkages were identified in both fractions, namely terminal D-glucopyranose (T-Glc*p*), terminal D-galactopyranose (T-Gal*p*), →4)-Glc*p*-(1→, →4)-Gal*p*-(1→, and →4,6)-Glc*p*-(1→. Among these, →4)-Glc*p*-(1→ was the predominant linkage in both fractions, whereas CP2 contained a higher proportion of →4,6)-Glc*p*-(1→ and terminal sugar residues than CP1. The observed linkage profiles were consistent with the monosaccharide composition presented in the previous section.

The presence of →4,6)-Glc*p*-(1→ residues together with terminal Glc*p* and Gal*p* residues is consistent with branching involving C-6 of some glucopyranosyl residues. CP2 contained a higher relative proportion of →4,6)-Glc*p*-(1→ than CP1, suggesting differences in the branching characteristics of the two fractions. However, methylation–GC–MS alone does not establish the anomeric configuration, residue sequence, or complete backbone architecture. Therefore, the present results are interpreted as predominant glycosidic linkage patterns and putative branching features, rather than a definitive primary structure. Further one- and two-dimensional NMR analyses would be required to establish the detailed molecular architecture of CP1 and CP2. Differences in linkage distribution and branching characteristics may be associated with variations in physicochemical and biological properties of polysaccharides [35]; however, the contribution of individual structural features to the observed antioxidant and macrophage-stimulating activities cannot be established from the present data alone.

### 3.5. Impact of Coconut Residue–Derived Polysaccharides on Cell Viability and NO Production in RAW264.7 Cells

**Figure 3:**
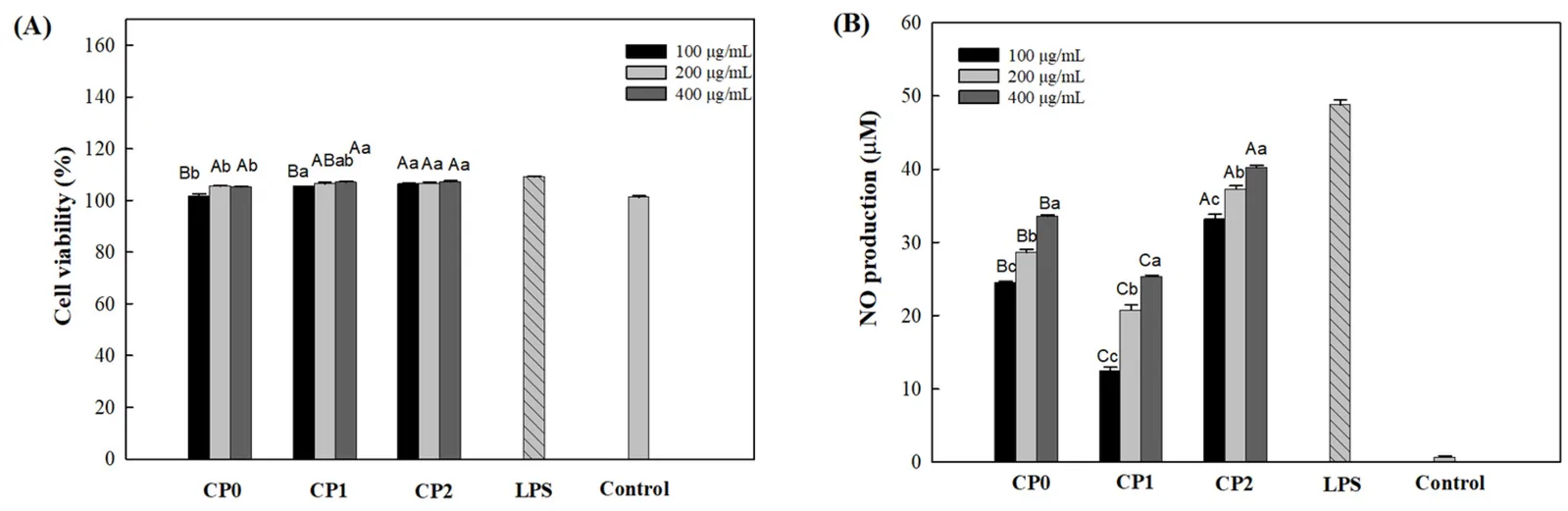
Effects of crude polysaccharide (CP0) and its purified fractions (CP1 and CP2) on (**A**) cell proliferation and (**B**) NO production in RAW264.7 cells. The culture medium alone served as a control, while LPS was used as a positive control. Data are expressed as mean ± SD (*n* = 3). Different uppercase letters indicate significant differences among concentrations within the same polysaccharide fraction, whereas different lowercase letters indicate significant differences among polysaccharide fractions at the same concentration (*p* < 0.05, two-way ANOVA followed by Tukey’s multiple-comparison test).

Prior to the macrophage experiments, endotoxin contamination of the polysaccharide preparations was evaluated using the LAL assay. No firm gel formation was observed for CP0, CP1, or CP2, whereas the endotoxin-positive control showed the expected gel formation. These results indicate that detectable endotoxin contamination was not observed in the tested polysaccharide preparations under the conditions of the assay. The effects of CP0 and its purified fractions (CP1 and CP2) on RAW264.7 macrophages are presented in Figure 3. All polysaccharide fractions exhibited no cytotoxicity at concentrations of 100–400 µg/mL, with cell viability exceeding 100% relative to the untreated control (Figure 3A). A slight increase in cell proliferation was observed after treatment with all fractions, although no significant differences were detected among the treatments at the same concentration. These results indicate that CP0, CP1, and CP2 possess good biocompatibility and are suitable for subsequent immunomodulatory evaluation.

The immunomodulatory activity of the polysaccharides was further assessed by measuring nitric oxide (NO) production (Figure 3B). All fractions stimulated NO production in a concentration-dependent manner; however, their activities differed significantly. CP2 exhibited the highest NO-stimulating response, producing significantly higher NO levels than CP0 and CP1 at all tested concentrations (*p* < 0.05). In contrast, CP1 showed the lowest activity (12.51–25.37 μM nitrite), whereas CP0 induced moderate NO production (24.48–33.55 μM). At the highest concentration, CP2 induced 40.25 μM nitrite, corresponding to approximately 82.5% of the response elicited by the LPS-treated group (48.76 μM).

Nitric oxide is a key mediator produced by activated macrophages and plays important roles in innate immunity, inflammatory responses, and host defense mechanisms [36]. Among the tested fractions, CP2 exhibited the highest NO-stimulating activity and also differed from CP0 and CP1 in several physicochemical characteristics, including molecular weight, molecular-weight distribution, monosaccharide composition, and branching profile. These concurrent differences suggest an association between the physicochemical characteristics of the polysaccharide fractions and their macrophage responses [37,38]. However, because multiple structural and compositional parameters differed simultaneously among the fractions, the contribution of any individual characteristic to the observed biological response cannot be established from the present data.

### 3.6. Radical-Scavenging Activity of CPs

**Figure 4:**
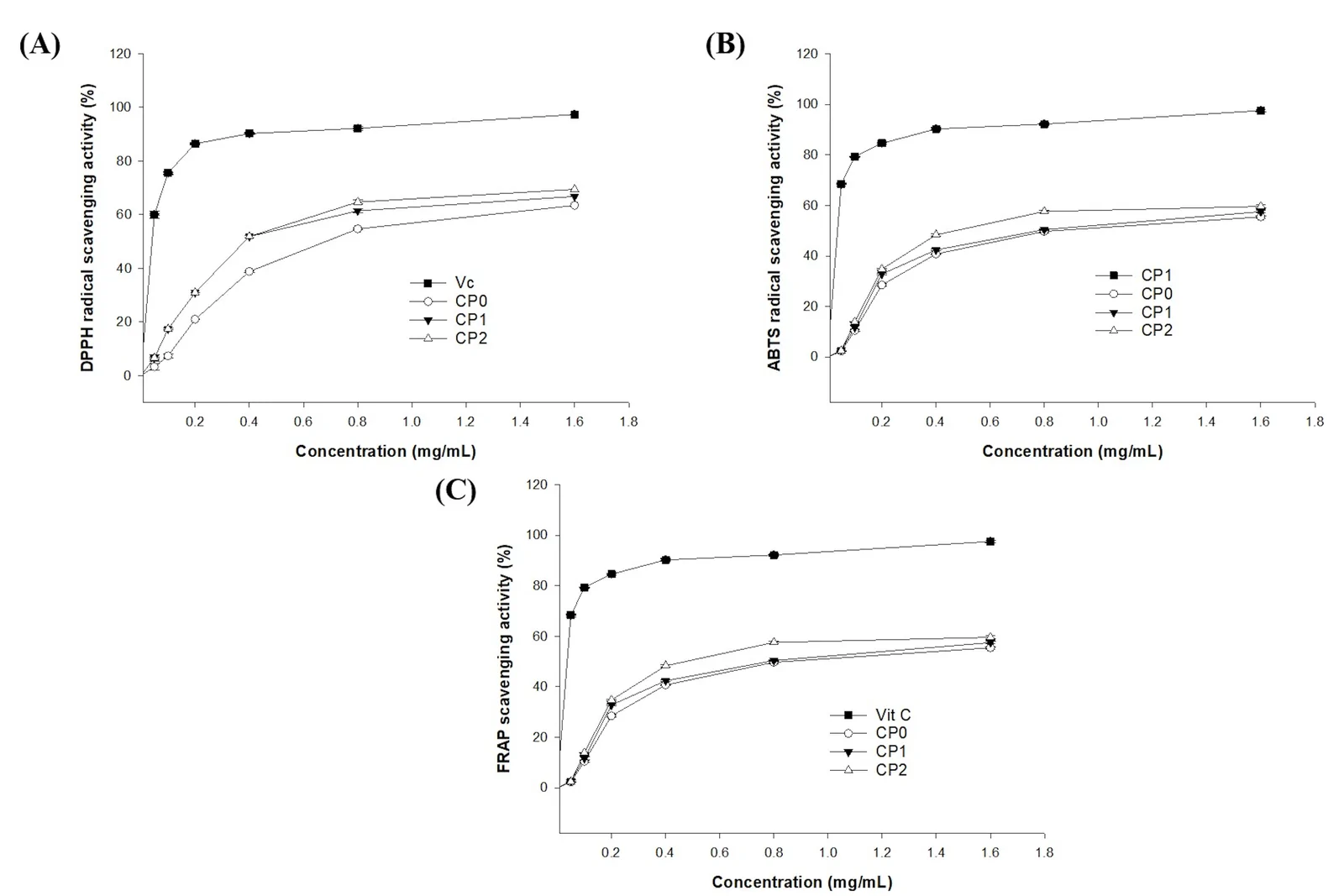
Radical-scavenging activities of coconut residue polysaccharide fractions (CP0, CP1, and CP2) compared with vitamin C (Vc). (**A**) DPPH radical scavenging activity, (**B**) ABTS radical scavenging activity, and (**C**) superoxide (O_2_•^−^) radical scavenging activity. All samples exhibited concentration-dependent radical-scavenging activities. Data are presented as mean ± SD (*n* = 3).

The radical-scavenging activities of coconut residue polysaccharides (CP0, CP1, and CP2) were evaluated using DPPH, ABTS, and superoxide radical scavenging assays over a concentration range of 0–1.6 mg/mL (Figure 4). Vitamin C (Vc) was included as a conventional positive reference for radical-scavenging activity. All polysaccharide fractions exhibited concentration-dependent increases in radical-scavenging activity. The calculated IC_50_ values and corresponding 95% confidence intervals (CI) are provided in Table S2.

In the DPPH assay (Figure 4A), scavenging activity increased progressively with concentration. At 0.1 mg/mL, CP0, CP1, and CP2 exhibited activities of 7.3–17.3%, which increased to 63.5%, 66.8%, and 69.5%, respectively, at 1.6 mg/mL. CP2 showed the lowest IC_50_ value (0.382 mg/mL; 95% CI: 0.376–0.388), followed by CP1 (0.431 mg/mL; 95% CI: 0.400–0.462) and CP0 (0.682 mg/mL; 95% CI: 0.609–0.754). Vc exhibited substantially greater activity, with an IC_50_ of 0.042 mg/mL.

A similar concentration-dependent response was observed in the ABTS assay (Figure 4B). At 1.6 mg/mL, CP2 reached approximately 59.6% scavenging activity, compared with approximately 55.4–57.5% for CP0 and CP1. The corresponding IC_50_ values were 0.470 mg/mL (95% CI: 0.444–0.496) for CP2, 0.778 mg/mL (95% CI: 0.747–0.810) for CP1, and 0.845 mg/mL (95% CI: 0.632–1.058) for CP0, confirming the greater ABTS radical-scavenging capacity of CP2 among the polysaccharide fractions. Vc showed considerably greater activity, with an IC_50_ of 0.037 mg/mL.

In the superoxide radical scavenging assay (Figure 4C), CP0, CP1, and CP2 also exhibited concentration-dependent responses, reaching approximately 62.7%, 67.0%, and 70.6%, respectively, at 1.6 mg/mL. However, their IC_50_ values were relatively similar, at 0.825 mg/mL (95% CI: 0.668–0.982) for CP0, 0.841 mg/mL (95% CI: 0.756–0.925) for CP1, and 0.802 mg/mL (95% CI: 0.630–0.975) for CP2. Thus, although CP2 showed the highest scavenging percentage at the highest tested concentration, the IC_50_ estimates indicate broadly comparable superoxide radical-scavenging capacities among the three polysaccharide fractions. Vc showed substantially greater activity, with an IC_50_ of 0.054 mg/mL.

Therefore, coconut residue polysaccharides demonstrated measurable in vitro chemical radical-scavenging capacity, with CP2 showing greater DPPH and ABTS scavenging capacity than CP0 and CP1, whereas the differences in superoxide scavenging capacity were less pronounced. Similar radical-scavenging properties have been reported for coconut-derived polysaccharides [7,8]. However, the observed differences among CP0, CP1, and CP2 cannot be attributed to individual structural characteristics based on the present data alone. Furthermore, these chemical radical-scavenging assays do not establish antioxidant efficacy in food matrices or biological systems, and further studies using appropriate food-based and biologically relevant models are warranted.

### 3.7. Changes in CP2 During Simulated Salivary, Gastric, and Intestinal Digestion

**Figure 5:**
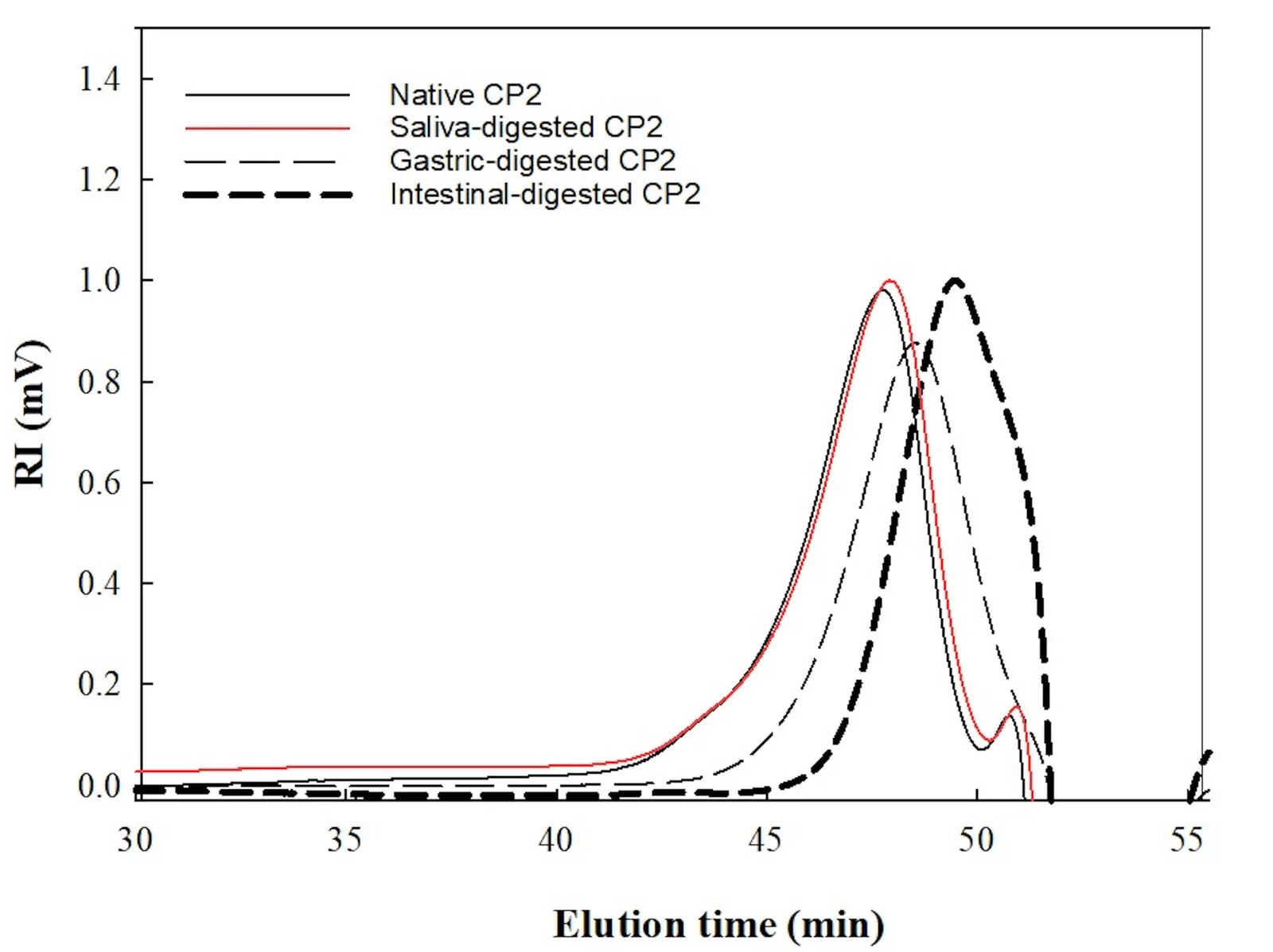
HPSEC chromatograms of native CP2 and CP2 after simulated gastrointestinal digestion.

Among the purified polysaccharides, CP2 exhibited the strongest macrophage-stimulating activity, particularly in terms of NO production in RAW264.7 macrophages. Therefore, CP2 was selected for further investigation of changes in its molecular characteristics during simulated gastrointestinal digestion. Changes in molecular weight (Mw) and monosaccharide composition are summarized in Table 4, while the corresponding HPSEC chromatograms are shown in Figure 5. Native CP2 exhibited an initial Mw of 72.6 ± 1.5 kDa, which decreased only slightly during the salivary phase (71.8–70.5 kDa), indicating relatively limited changes in apparent molecular size. This observation is consistent with previous reports showing that many plant-derived non-starch polysaccharides undergo relatively limited changes during simulated oral digestion [39,40,41].

In contrast, a pronounced reduction in Mw was observed during the gastric phase, decreasing from 65.3 ± 1.6 kDa at 5 min to 44.2 ± 1.6 kDa after 120 min. During the intestinal phase, Mw further decreased from 42.8 ± 1.3 kDa to 33.1 ± 1.5 kDa, indicating a progressive shift toward smaller apparent molecular sizes. Acidic gastric conditions and the physicochemical environment of the intestinal phase may contribute to these changes, as previously reported for other plant-derived polysaccharides [36,42,43,44,45]. However, because identically processed non-digested controls were not included, contributions from sample-processing steps, including pH adjustment, heating, centrifugation, dialysis, and freeze-drying, cannot be excluded. Therefore, the observed changes are interpreted as digestion-associated changes in molecular-size distribution rather than direct evidence of glycosidic-bond hydrolysis.

The HPSEC chromatograms were consistent with these observations, showing a gradual shift in the major peak toward longer elution times following simulated digestion (Figure 5), consistent with a reduction in apparent molecular size. Slight changes in relative monosaccharide composition were also observed. The glucose content decreased from 39.5% to 36.0%, whereas the relative galactose content increased from 60.5% to 64.0%, resulting in an increase in the Gal/Glc ratio from 1.53 to 1.78. These compositional changes indicate alterations in the relative monosaccharide distribution following digestion and processing; however, without complementary mass-balance or reducing-sugar data, preferential hydrolysis of specific glucose-containing linkages cannot be conclusively established.

Overall, CP2 showed relatively limited changes in apparent molecular size during the salivary phase, whereas progressively lower Mw values were observed following gastric and intestinal digestion. These molecular changes occurred concurrently with changes in the radical-scavenging and macrophage responses of CP2, suggesting an association between digestion-associated molecular changes and biological responses rather than establishing a direct causal relationship. Because process-matched controls, stage-specific recovery yields, reducing-sugar measurements, and complete mass-balance analyses were not included in the original experimental design, the relative contributions of gastrointestinal conditions and post-digestion processing cannot be distinguished. Further studies incorporating these controls will be required to establish the mechanisms underlying the observed molecular changes.

### 3.8. Macrophage Responses to Digested CP2

#### 3.8.1. Cell Viability and NO Production

**Figure 6:**
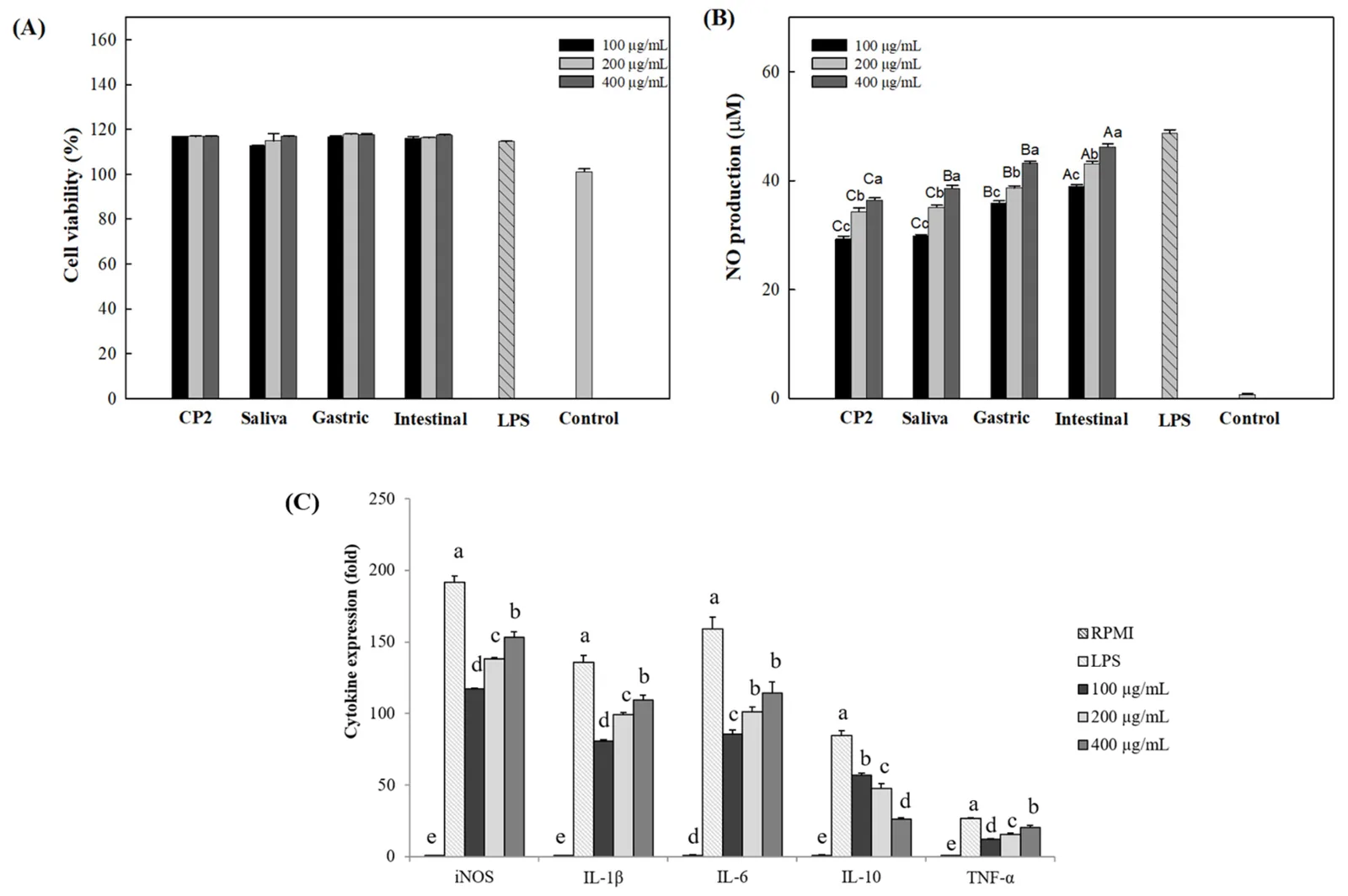
Effects of digested CP2 on macrophage responses in RAW264.7 cells. (**A**) Cell viability of RAW264.7 macrophages treated with native CP2 and saliva-, gastric-, and intestinal-digested CP2 at 100, 200, and 400 µg/mL. Data are presented as mean ± SD (n = 3). (**B**) NO production in RAW264.7 macrophages treated with native and digested CP2. Different uppercase letters indicate significant differences among digestion treatments at the same concentration, whereas different lowercase letters indicate significant differences among concentrations within the same treatment (*p* < 0.05; two-way ANOVA followed by Tukey’s multiple-comparison test). (**C**) Relative mRNA expression of *iNOS*, *IL-1β*, *IL-6*, *IL-10*, and *TNF-α* in RAW264.7 macrophages treated with intestinal-digested CP2 at 100, 200, and 400 µg/mL. LPS and RPMI were used as positive and untreated controls, respectively. Different letters indicate significant differences among treatments within each gene (*p* < 0.05; one-way ANOVA followed by Tukey’s multiple-comparison test). Data are presented as mean ± SD (n = 3).

Based on its greater macrophage-stimulating activity before digestion, CP2 was selected for simulated salivary, gastric, and intestinal digestion. The viability of RAW264.7 cells exposed to native and digested CP2 was evaluated at concentrations of 100–400 µg/mL using the WST assay. Treatment with native, saliva-, gastric-, and intestinal-digested CP2 did not reduce cell viability relative to the untreated control, with viability remaining above 100% under all tested conditions (Figure 6A). Therefore, concentrations of 100, 200, and 400 µg/mL were used for subsequent experiments.

No firm gel formation was observed for the intestinal-digested CP2 preparation in the LAL assay, whereas the endotoxin-positive control showed the expected gel formation. These results indicate that detectable endotoxin contamination was not observed in the intestinal-digested CP2 preparation under the conditions of the assay. Macrophage activation was further assessed by measuring nitric oxide (NO) production (Figure 6B). LPS stimulation significantly increased NO production (48.67 ± 0.70 µM) compared with the untreated control (0.69 ± 0.18 µM). NO production increased with increasing concentrations of the CP2 preparations. Saliva-digested CP2 induced 29.27–36.41 µM nitrite, whereas gastric- and intestinal-digested CP2 produced 29.83–38.55 µM and 38.85–46.18 µM nitrite, respectively. Among the recovered preparations, intestinal-digested CP2 was associated with the highest NO production, reaching approximately 94.9% of the LPS response at 400 µg/mL.

Nitric oxide is an important mediator produced by activated macrophages and participates in innate immune defense and inflammatory regulation [36]. The higher NO production observed in macrophages treated with gastric- and intestinal-digested CP2 occurred concurrently with the reduction in apparent molecular weight observed during simulated digestion. These findings indicate an association between digestion-associated molecular changes and macrophage responses rather than establishing a direct causal relationship. Because specific macrophage receptors and intracellular signaling pathways were not directly investigated, the molecular mechanism underlying this response cannot be established from the present data. Furthermore, although digestive-fluid controls were included during the digestion procedure, fully process-matched digestive-fluid blanks subjected to the complete post-digestion processing sequence and subsequent cell assays were not evaluated. Therefore, potential contributions from residual digestion components or sample-processing effects cannot be completely excluded. Intestinal-digested CP2, which produced the highest NO response among the recovered preparations, was subsequently selected for evaluation of macrophage-related gene expression.

#### 3.8.2. Stimulating Effect of Intestinal-Digested CP2 on mRNA Expression

To further characterize the macrophage response to CP2 following gastrointestinal digestion, the mRNA expression of *iNOS*, *TNF-α*, *IL-1β*, *IL-6*, and *IL-10* was determined in RAW264.7 macrophages (Figure 6C). Intestinal-digested CP2 significantly upregulated the expression of *iNOS*, *TNF-α*, *IL-1β*, and *IL-6*, while *IL-10* expression was significantly reduced compared with the untreated control (*p* < 0.05). Within the concentration range of 100–400 µg/mL, the expression of all pro-inflammatory markers increased in a concentration-dependent manner, with the highest levels observed at 400 µg/mL, although the responses remained lower than those induced by LPS. These findings indicate that intestinal-digested CP2 induces a predominantly pro-inflammatory macrophage response under the tested conditions.

Activated macrophages regulate innate immunity through the production of inflammatory mediators, including iNOS, TNF-α, IL-1β, and IL-6, which are characteristic markers of M1-like macrophage activation [46]. In contrast, IL-10 is an anti-inflammatory cytokine that contributes to the regulation of excessive inflammatory responses [47]. The increased expression of pro-inflammatory mediators together with the reduction in IL-10 observed in the present study suggests that intestinal-digested CP2 favors a pro-inflammatory macrophage phenotype. Similar macrophage-stimulating responses have been reported for digested polysaccharides from *Grifola frondosa* [48] and *Auricularia cornea* [49]. In the present study, increased NO production and pro-inflammatory gene expression following digestion occurred concurrently with a reduction in the apparent molecular weight of CP2, suggesting an association between digestion-associated molecular changes and macrophage responses rather than a direct causal relationship.

Several pattern-recognition receptors, including Toll-like receptor 4 (TLR4), Dectin-1, and the mannose receptor (CD206), have been reported to participate in macrophage recognition of polysaccharides, depending on their structural characteristics. These receptors may therefore represent potential candidates involved in the macrophage response to intestinal-digested CP2. However, receptor-binding or blocking experiments were not performed, and the involvement of any specific receptor cannot be established from the current data. Similarly, although TLR-mediated MAPK and NF-κB signaling pathways have been implicated in macrophage activation by other polysaccharides, these pathways were not directly investigated. Therefore, the involvement of these receptors and downstream signaling pathways in the response to intestinal-digested CP2 remains a proposed mechanism requiring further experimental validation.

Moreover, the observed increase in pro-inflammatory mediators together with reduced IL-10 expression should not be interpreted as unequivocally beneficial immune enhancement, as excessive or sustained pro-inflammatory macrophage activation may be undesirable. Further studies evaluating secreted cytokine levels, phagocytic activity, macrophage polarization markers, receptor-binding or blocking experiments, downstream signaling pathways, and a broader range of safety endpoints are required to establish the physiological relevance and safety of CP2 for potential food applications.

### 3.9. Radical-Scavenging Activity of Digested CP2

**Figure 7:**
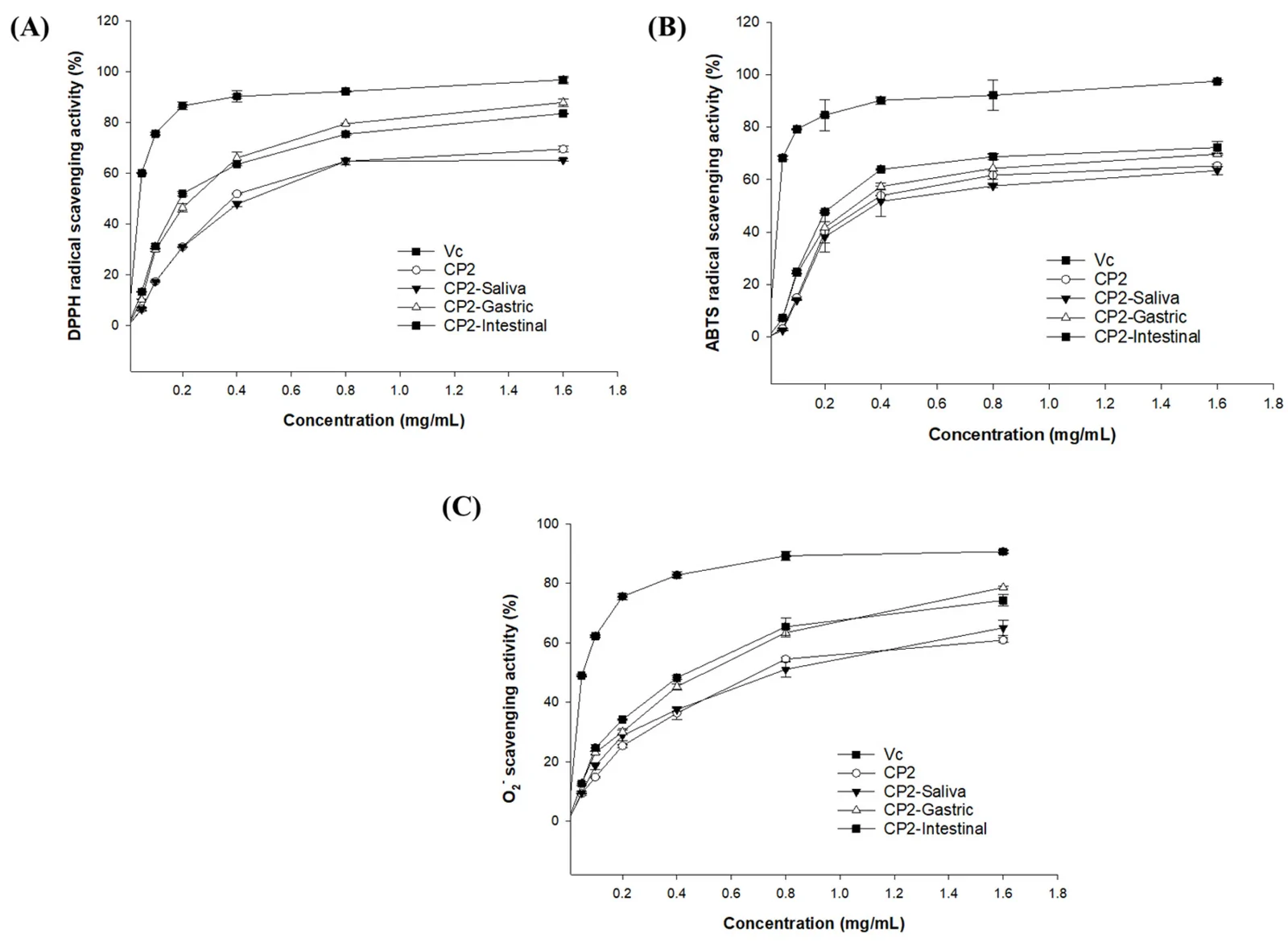
Effects of simulated gastrointestinal digestion on the radical-scavenging activities of CP2. (**A**) DPPH, (**B**) ABTS, and (**C**) superoxide (O_2_•^-^) radical scavenging activities of native and digested CP2 (salivary-, gastric-, and intestinal-digested samples) compared with vitamin C (Vc). Data are presented as mean ± SD (*n* = 3).

The radical-scavenging activities of CP2 following simulated salivary, gastric, and intestinal digestion were evaluated using DPPH, ABTS, and superoxide radical scavenging assays over a concentration range of 0–1.6 mg/mL (Figure 7). Native CP2 was included as the undigested reference, while vitamin C (Vc) was included as a conventional positive reference for radical-scavenging activity. All samples exhibited concentration-dependent increases in radical-scavenging activity. The calculated IC_50_ values and corresponding 95% confidence intervals (CI) are presented in Table S2.

In the DPPH assay (Figure 7A), gastric- and intestinal-digested CP2 generally exhibited greater scavenging activity than native and saliva-digested CP2. The IC_50_ decreased from 0.382 mg/mL (95% CI: 0.376–0.388) for native CP2 to 0.235 mg/mL (95% CI: 0.207–0.263) and 0.190 mg/mL (95% CI: 0.180–0.201) following gastric and intestinal digestion, respectively. In contrast, saliva-digested CP2 exhibited an IC_50_ of 0.451 mg/mL (95% CI: 0.393–0.508). These results indicate greater DPPH radical-scavenging capacity following gastric and intestinal digestion.

A similar pattern was observed in the ABTS assay (Figure 7B). Native CP2 exhibited an IC_50_ of 0.344 mg/mL (95% CI: 0.331–0.356), whereas gastric- and intestinal-digested CP2 showed lower IC_50_ values of 0.299 mg/mL (95% CI: 0.228–0.370) and 0.228 mg/mL (95% CI: 0.210–0.246), respectively. Saliva-digested CP2 exhibited an IC_50_ of 0.405 mg/mL (95% CI: 0.127–0.682). Among the digested samples, intestinal-digested CP2 therefore showed the greatest ABTS radical-scavenging capacity.

In the superoxide radical scavenging assay (Figure 7C), the IC_50_ values of native and saliva-digested CP2 were 0.700 mg/mL (95% CI: 0.641–0.758) and 0.783 mg/mL (95% CI: 0.617–0.949), respectively. Gastric and intestinal digestion resulted in lower IC_50_ values of 0.506 mg/mL (95% CI: 0.451–0.560) and 0.442 mg/mL (95% CI: 0.405–0.478), respectively, indicating greater superoxide radical-scavenging capacity after these digestion phases.

Therefore, the radical-scavenging properties of CP2 were influenced following simulated gastrointestinal digestion. Salivary digestion produced relatively minor changes, whereas gastric and intestinal digestion were generally associated with lower IC_50_ values and greater radical-scavenging capacity. Previous studies have similarly reported changes in the radical-scavenging properties of polysaccharides following simulated gastrointestinal digestion [16,50]. In the present study, these changes occurred concurrently with the reduction in molecular weight of CP2 following digestion and processing, suggesting an association between digestion-associated molecular changes and radical-scavenging capacity rather than establishing a direct causal relationship. Further studies using appropriate digestion-process controls, food matrices, and biologically relevant antioxidant models are required to determine whether these in vitro chemical radical-scavenging effects translate into antioxidant efficacy under physiological or food-system conditions.

Moreover, although the present findings demonstrate in vitro macrophage-stimulating and radical-scavenging properties of CP2, these results alone are insufficient to establish its practical efficacy as a functional food ingredient. The present study did not evaluate food-matrix compatibility, sensory acceptability, storage stability, feasible dietary intake, intestinal absorption, comprehensive safety, or in vivo efficacy. Colonic fermentation and prebiotic activity were also not evaluated; therefore, the potential effects of CP2 on gut microbiota and prebiotic function remain to be established. Furthermore, although the recovery of polysaccharides from coconut residue represents a potential value-added utilization pathway, its techno-economic feasibility and environmental benefits were not directly assessed. The absence of a polysaccharide-based reference is an additional limitation of the present antioxidant evaluation, as vitamin C was used only as a conventional positive reference rather than as a structurally comparable control. Future studies should therefore investigate the fermentability of CP2, its effects on beneficial gut bacteria and short-chain fatty acid production, and include an appropriate polysaccharide comparator together with food-matrix, safety, in vivo*,* techno-economic, and environmental evaluations before its practical food applications and sustainability benefits can be established.

## 4. Conclusions

In conclusion, polysaccharides were successfully extracted and purified from coconut residue, yielding galactose- and glucose-rich fractions with distinct molecular characteristics. Among the purified fractions, CP2 exhibited the lowest molecular weight and showed greater in vitro DPPH and ABTS radical-scavenging capacity and higher NO production in RAW264.7 macrophages without reducing cell viability. Following simulated gastrointestinal digestion, CP2 exhibited a progressive reduction in apparent molecular weight and slight changes in relative monosaccharide composition, which occurred concurrently with changes in radical-scavenging capacity and macrophage responses. Intestinal-digested CP2 produced the highest NO response among the recovered preparations, together with increased expression of *iNOS*, *TNF-α*, *IL-1β*, and *IL-6* and reduced *IL-10* expression. However, these findings indicate associations between digestion-associated molecular changes and biological responses rather than a direct causal structure–activity relationship. Overall, CP2 represents a bioactive polysaccharide fraction warranting further investigation for potential food-related applications and value-added utilization of coconut residue. Further studies incorporating appropriate process controls, safety and in vivo evaluations, and food-matrix, techno-economic, and environmental assessments are required before its practical application can be established.

## Figures and Tables

**Table 1:** Chemical composition and monosaccharide content of crude polysaccharide (CP0) and purified fractions (CP1 and CP2) from coconut residue.

Chemical Composition	CP0	CP1	CP2
Carbohydrate (%)	96.7 ± 0.3 ^b^	97.1 ± 0.2 ^a^	97.7 ± 0.5 ^a^
Protein (%)	2.90 ± 0.0 ^a^	2.90 ± 0.1 ^a^	2.30 ± 0.2 ^b^
Monosaccharide content (%)			
D-Glucose	45.1 ± 0.0 ^b^	48.3 ± 0.1 ^a^	39.5 ± 0.3 ^c^
D-Galactose	54.9 ± 0.0 ^b^	51.7 ± 0.3 ^c^	60.5 ± 0.21 ^a^

Different letters indicate significant differences (*p* < 0.05) among the groups in each row for total carbohydrate and monosaccharide content.

**Table 2:** Molecular weight characteristics of crude (CP0) and purified polysaccharide fractions (CP1 and CP2) from coconut residue.

Samples	Mw (kDa)	Rg (nm)	Mn (kDa)	PDI (Mw/Mn)
CP0	369.2 ± 2.84 ^b^	58.8 ± 0.8 ^b^	242.5 ± 0.24 ^a^	1.52 ^a^
CP1	455.8 ± 0.55 ^a^	54.7 ± 4.3 ^b^	215.2 ± 0.21 ^a^	2.12 ^a^
CP2	72.6 ± 0.85 ^b^	64.5 ± 0.5 ^a^	66.5 ± 0.06 ^b^	1.35 ^b^

Different letters indicate significant differences (*p* < 0.05) among the groups in each column for molecular properties.

**Table 3:** Glycosidic linkage composition of purified polysaccharide fractions (CP1 and CP2) as determined by methylation analysis.

Samples	Retention Time (Min)	Methylation Product	Glycosidic Linkage	Peak Area (%)
**CP1**	9.18	1,5-di-O-acetyl-2,3,4,6-tetra-O-methyl-Glc	Glc*p*-(1→	11.2
	9.49	1,5-di-O-acetyl-2,3,4,6-tetra-O-methyl-Gal	Gal*p*-(1→	2.3
	12.42	1,4,5-tri-O-acetyl-2,3,6-tri-O-methyl-Glc	→4)-Glc*p*-(1→	52.5
	12.67	1,4,5-tri-O-acetyl-2,3,6-tri-O-methyl-Gal	→4)-Gal*p*-(1→	20.0
	13.95	1,4,5,6- tetra-O-acetyl-2,3-di-O-methyl-Glc	→4,6)-Glc*p*-(1→	14.0
**CP2**	9.18	1,5-di-O-acetyl-2,3,4,6-tetra-O-methyl-Glc	Glc*p*-(1→	15
	9.49	1,5-di-O-acetyl-2,3,4,6-tetra-O-methyl-Gal	Gal*p*-(1→	5.0
	12.42	1,4,5-tri-O-acetyl-2,3,6-tri-O-methyl-Glc	→4)-Glc*p*-(1→	47.2
	12.67	1,4,5-tri-O-acetyl-2,3,6-tri-O-methyl-Gal	→4)-Gal*p*-(1→	12.0
	13.95	1,4,5,6- tetra-O-acetyl-2,3-di-O-methyl-Glc	→4,6)-Glc*p*-(1→	20.8

**Table 4:** Changes in molecular weight and relative monosaccharide profile of CP2 during simulated gastrointestinal digestion.

Digestion Stage	Time	Mw (kDa)	Glc (%)	Gal (%)	Gal/Glc Ratio
Native	–	72.6 ± 1.5	39.5 ± 0.3	60.5 ± 0.2	1.53
Saliva	5 min	71.8 ± 1.3	39.4 ± 0.2	60.6 ± 0.2	1.54
	15 min	71.2 ± 1.4	39.2 ± 0.3	60.8 ± 0.3	1.55
	30 min	70.5 ± 1.2	39.1 ± 0.2	60.9 ± 0.2	1.56
Gastric	5 min	65.3 ± 1.6	38.7 ± 0.3	61.3 ± 0.3	1.58
	15 min	60.8 ± 1.8	38.4 ± 0.2	61.6 ± 0.2	1.60
	30 min	55.6 ± 1.5	38.0 ± 0.3	62.0 ± 0.3	1.63
	60 min	49.7 ± 1.4	37.7 ± 0.2	62.3 ± 0.2	1.65
	120 min	44.2 ± 1.6	37.4 ± 0.3	62.6 ± 0.3	1.67
Intestinal	5 min	42.8 ± 1.3	37.2 ± 0.2	62.8 ± 0.2	1.69
	15 min	40.5 ± 1.2	36.9 ± 0.3	63.1 ± 0.3	1.71
	30 min	38.2 ± 1.4	36.6 ± 0.2	63.4 ± 0.2	1.73
	60 min	35.6 ± 1.6	36.3 ± 0.3	63.7 ± 0.3	1.75
	120 min	33.1 ± 1.5	36.0 ± 0.2	64.0 ± 0.2	1.78

## Data Availability

The original contributions presented in this study are included in the article/Supplementary Material. Further inquiries can be directed to the corresponding author.

## Supplementary Materials

The following supporting information can be downloaded at: https://www.mdpi.com/article/10.3390/foods15173105/s1, Figure S1: Monosaccharide profiles of standard monosaccharides and coconut residue polysaccharides (CP0, CP1, and CP2); Figure S2: GC–MS chromatograms of partially methylated alditol acetates (PMAAs) derived from CP1 and CP2; Figure S3: Mass spectra of partially methylated alditol acetates (PMAAs) identified in CP1 and CP2; Table S1: Primer sequences used for quantitative real-time PCR analysis; Table S2: IC_50_ values and 95% confidence intervals for DPPH, ABTS, and superoxide radical scavenging activities of coconut residue polysaccharides before and after simulated gastrointestinal digestion.
