# Supplementary material for: Extraction, Isolation, and Structural Characterization of Coconut Residue Polysaccharides: Immunomodulatory Activity, Antioxidant Capacity, and Gastrointestinal Digestive Behavior
Source: Foods. 2026 Sep 1;15(17):3105. doi: 10.3390/foods15173105 (PMC13565199; doi:10.3390/foods15173105)
Supplement: Supplementary file 1 [file foods-15-03105-s001.zip › foods-4526121-supplementary.pdf]

Supplementary data#

Figure

Figure S1. Monosaccharide profiles of (A) standard monosaccharides and (B) crude polysaccharide (CP0) and purified fractions CP1 and CP2 from coconut residue.

(A)

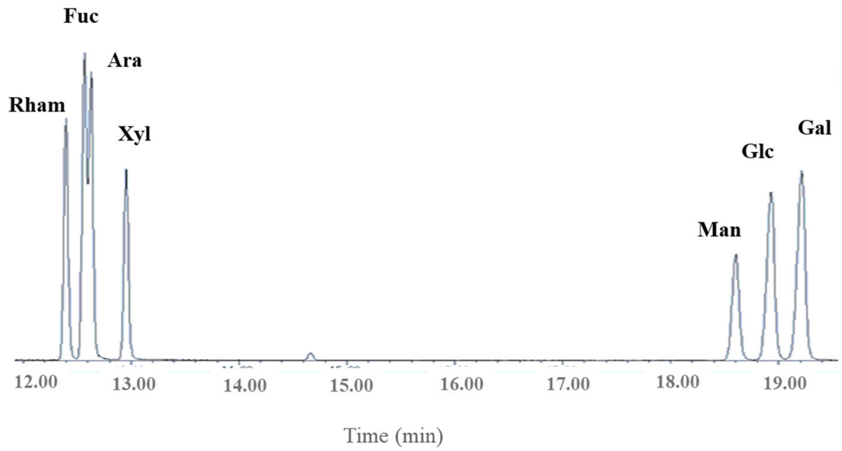

(B)

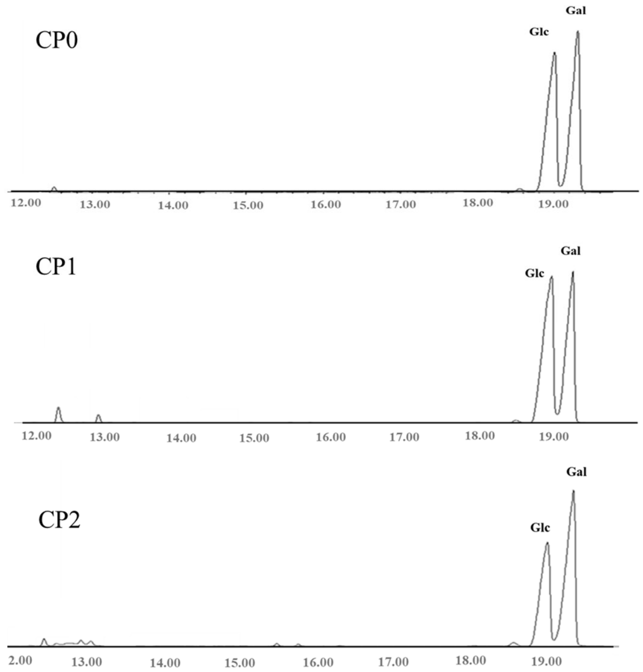

Figure S2. GC–MS chromatograms of partially methylated alditol acetates (PMAAs) derived from CP1 and CP2.

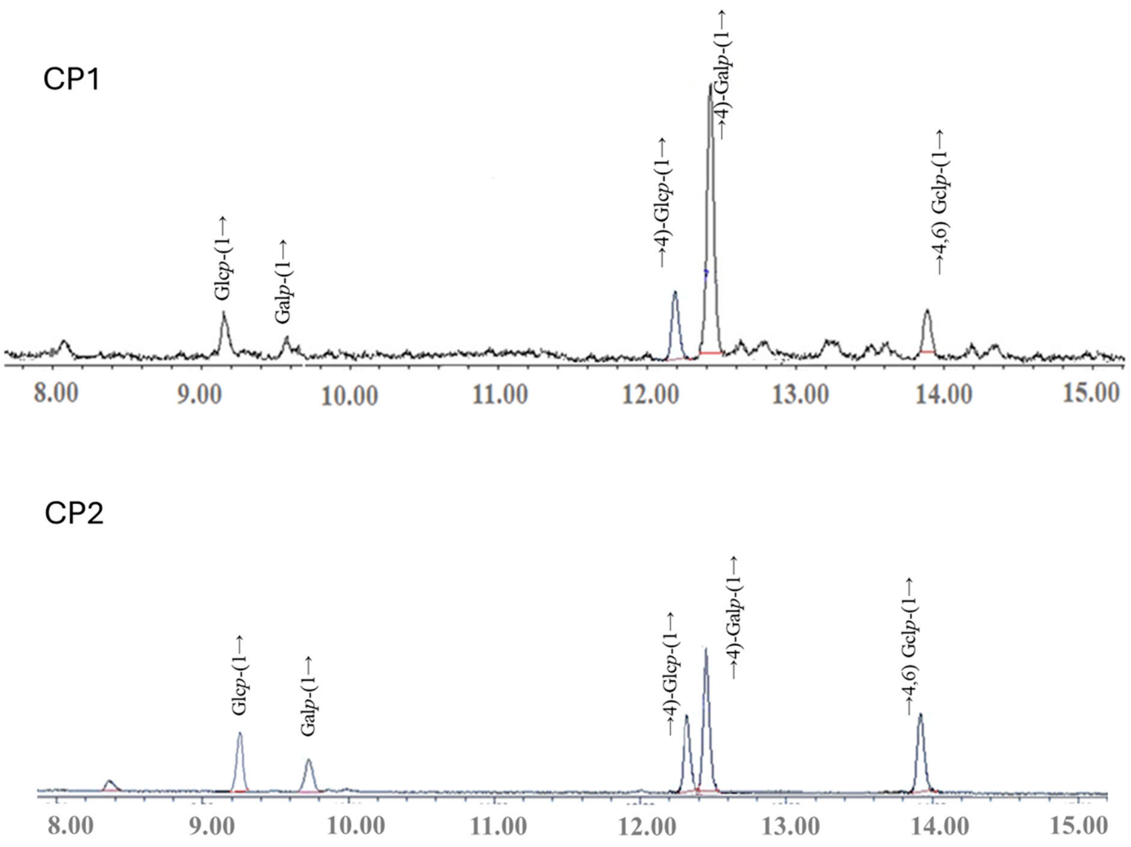

Figure S3. Mass spectrum of PMAAs

1,5-di-O-acetyl-2,3,4,6-tetra-O-methyl-Glc

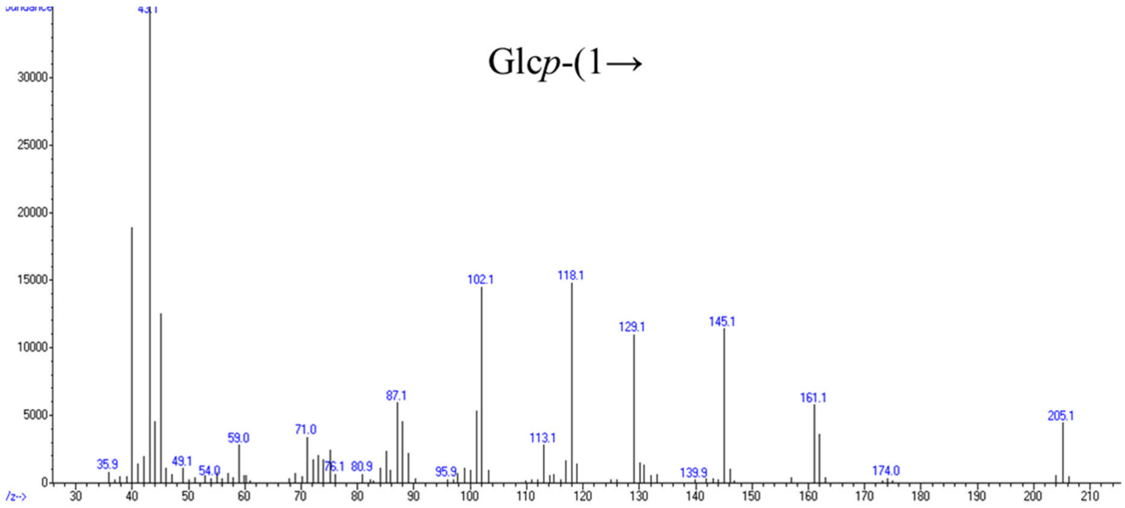

1,5-di-O-acetyl-2,3,4,6-tetra-O-methyl-Gal

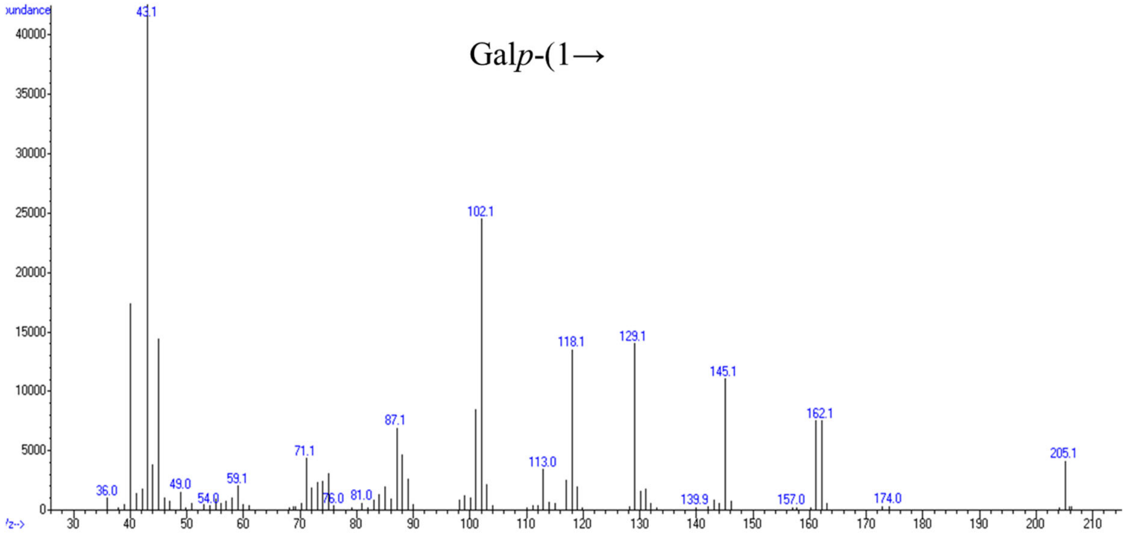

1,4,5-tri-O-acetyl-2,3,6-tri-O-methyl-Glc

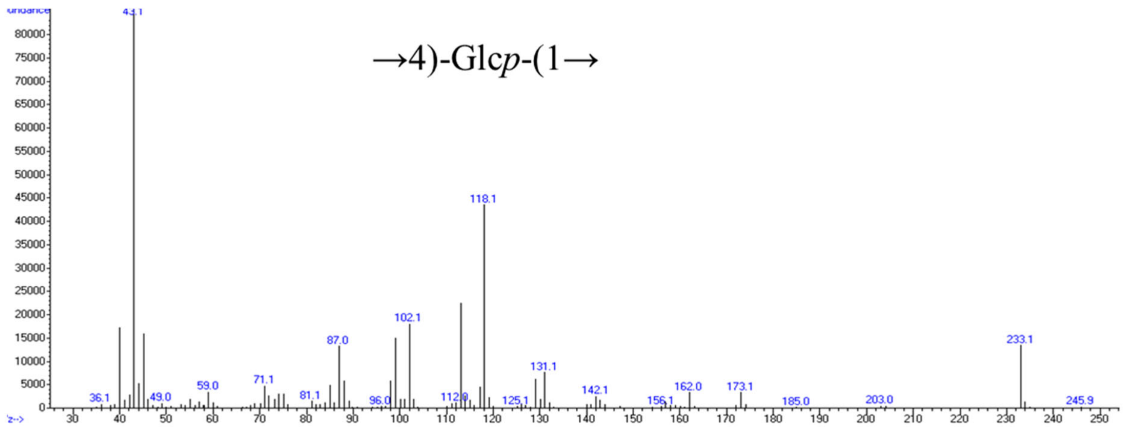

1,4,5-tri-O-acetyl-2,3,6-tri-O-methyl-Gal

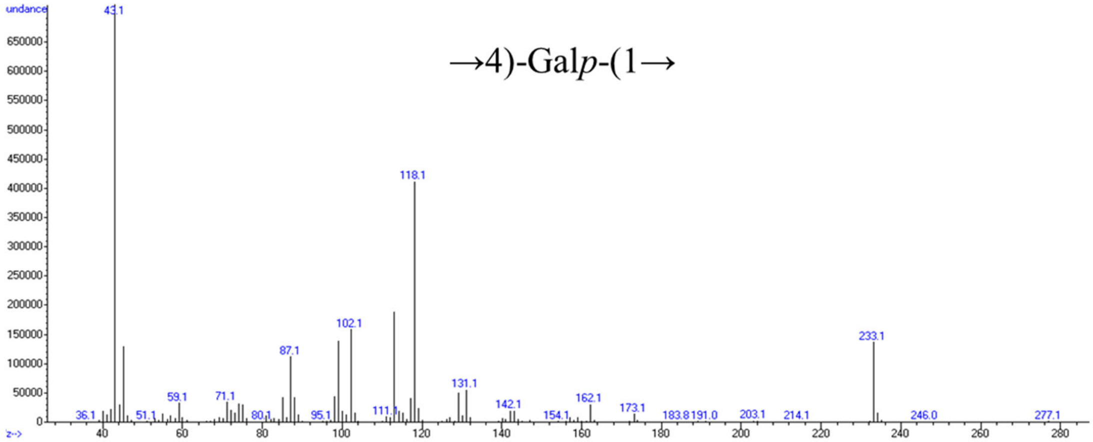

1,4,5,6-tetra-O-acetyl-2,3-di-O-methyl-Glc

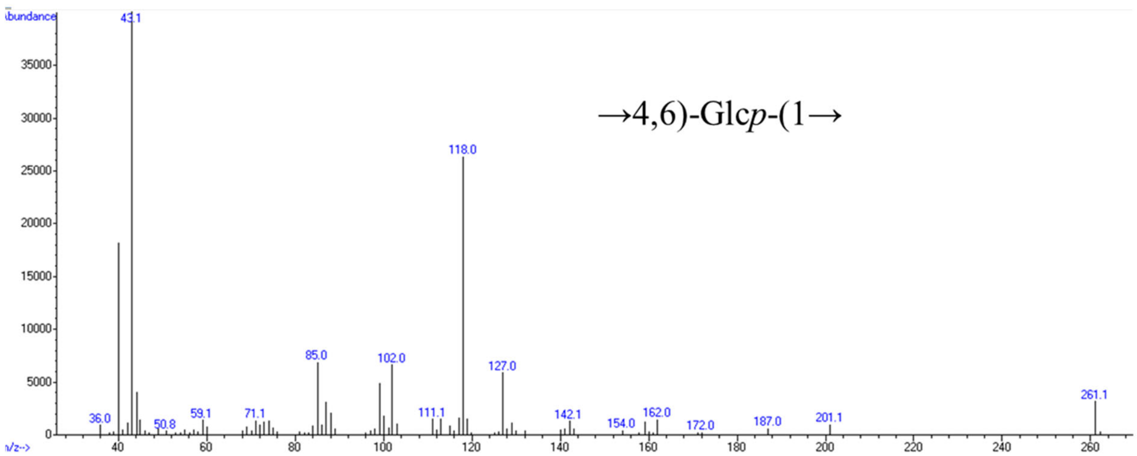

Table

Table S1. Primer sequences used for quantitative real-time PCR analysis.

**Gene**	**Forward primer (5′–3′)**	**Reverse primer (5′–3′)**
iNOS	TTCCAGAATCCCTGGACAAG	TGGTCGAAACTCTTGGGGTTTC
TNF-α	ATGAGCACAGAAAGCATGATC	TACAGGCTTGTCACTCGAATT
IL-1β	GGGCTCAAGGGAAAGAATC	TACCAGTTGGGGAACTCTGC
IL-6	AGTTGCCTTCTTGGGACTGA	CAGAATTGCCATTGCACAA
IL-10	TACCTGGTAGAAGTGATGCC	CATCATGTATGCTTCTATGC
β-actin	CCACAGCTGAGAGGGAAATC	AAGGAAGGCTGGAAAAGAGC

Table S2. IC_{50} values and 95% confidence intervals for DPPH, ABTS, and superoxide radical#scavenging activities of coconut residue polysaccharides before and after simulated gastrointestinal digestion.

Gljhvwlrq#vwdwxv#	Dvvd|#	Vdpsoh#	IF50#+pj2pO,	<8(#FL
Ehiruh#gljhvwlrq#	GSSK#	FS3#	319;5	3193<%31:87
		FS4#	31764	31733%31795
		FS5#	316;5	316:9%316;;
		Yf#	31375	31375%31375
	DEWV#	FS3#	31;78	31965%4138;
		FS4#	31::;	31:7:%31;43
		FS5#	317:3	31777%317<9
		Yf#	3136:	31369%3136:
	Vxshur{lgh#	FS3#	31;58	3199;%31<;5
		FS4#	31;74	31:89%31<58
		FS5#	31;35	31963%31<:8
		Yf#	31387	31386%31388
Diwhu#gljhvwlrq#	GSSK#	Qdwlyh#FS5#	316;5	316:9%316;;
		Vdolydu|0gljhvwhg#FS5#	31784	316<6%3183;
		Jdvwulf0gljhvwhg#FS5#	31568	3153:%31596
		Lqwhvwlqdo0gljhvwhg#FS5#	314<3	314;3%31534
	DEWV#	Qdwlyh#FS5#	31677	31664%31689
		Vdolydu|0gljhvwhg#FS5#	31738	3145:%319;5
		Jdvwulf0gljhvwhg#FS5#	315<<	3155;%316:3
		Lqwhvwlqdo0gljhvwhg#FS5#	3155;	31543%31579
	Vxshur{lgh#	Qdwlyh#FS5#	31:33	31974%31:8;
		Vdolydu|0gljhvwhg#FS5#	31:;6	3194:%31<7<
		Jdvwulf0gljhvwhg#FS5#	31839	31784%31893
		Lqwhvwlqdo0gljhvwhg#FS5#	31775	31738%317:;

#Values are expressed as IC_{50} (mg/mL) with corresponding 95% confidence intervals (CI), calculated from concentration–response data obtained from triplicate measurements (n = 3). Lower IC_{50} values indicate greater radical-scavenging activity. Vc, vitamin C.#
